# New Insights Into the Function of Flavohemoglobin in *Mycobacterium tuberculosis*: Role as a NADPH-Dependent Disulfide Reductase and D-Lactate-Dependent Mycothione Reductase

**DOI:** 10.3389/fcimb.2021.796727

**Published:** 2022-02-10

**Authors:** Naveen Thakur, Amar Nath Sharma, Mangesh Dattu Hade, Ajay Chhaya, Ashwani Kumar, Ravinder Singh Jolly, Kanak L. Dikshit

**Affiliations:** ^1^ CSIR-Institute of Microbial Technology, Chandigarh, India; ^2^ Department of Biotechnology, Panjab University, Chandigarh, India

**Keywords:** flavohemoglobin, *Mycobacterium tuberculosis*, FAD, D-lactate, thioredoxin reductase, oxidative stress

## Abstract

*Mycobacterium tuberculosis* (*Mtb*) produces an unconventional flavohemoglobin (*Mtb*FHb) that carries a FAD-binding site similar to D-lactate dehydrogenases (D-LDH) and oxidizes D-lactate into pyruvate. The molecular mechanism by which *Mtb*FHb functions in *Mtb* remains unknown. We discovered that the D-LDH-type FAD-binding site in *Mtb*FHb overlaps with another FAD-binding motif similar to thioredoxin reductases and reduces DTNB in the presence of NADPH similar to trxB of *Mtb*. These results suggested that *Mtb*FHb is functioning as a disulfide oxidoreductase. Interestingly, D-lactate created a conformational change in *Mtb*FHb and attenuated its ability to oxidize NADPH. Mass spectroscopy demonstrated that *Mtb*FHb reduces des-*myo-*inositol mycothiol in the presence of D-lactate unlike NADPH, indicating that D-lactate changes the specificity of *Mtb*FHb from di-thiol to di-mycothiol. When *M. smegmatis* carrying deletion in the *fhb*II gene (encoding a homolog of *Mtb*FHb) was complemented with the *fhb* gene of *Mtb*, it exhibited four- to fivefold reductions in lipid peroxidation and significant enhancement in the cell survival under oxidative stress. These results were corroborated by reduced lipid peroxidation and enhanced cell survival of wild-type *M. smegmatis* after overexpression of the *fhb* gene of *Mtb*. Since D-lactate is a by-product of lipid peroxidation and *Mtb*FHb is a membrane-associated protein, D-lactate-mediated reduction of mycothiol disulfide by *Mtb*FHb may uniquely equip *Mtb* to relieve the toxicity of D-lactate accumulation and protect the cell from oxidative damage, simultaneously balancing the redox environment under oxidative stress that may be vital for the pathogenesis of *Mtb*.

## Introduction

*Mycobacterium tuberculosis* (*Mtb*), the causative agent of tuberculosis, continues to be a major threat to human life, causing millions of deaths annually (Bloom and Murray, 1992; Dye, 2006). The extraordinary ability of the tubercle bacillus to survive within the intracellular environment of its host, where a highly toxic environment exists due to scarcity of oxygen and high levels of toxic reactive species, depends primarily on its multi-tier defense system and metabolic flexibility. Being an obligate aerobe, *Mtb* continually remains exposed to endogenous reactive oxygen as a part of normal aerobic respiration and additionally gets exposed to copious amounts of host-generated toxic reactive species during intracellular infection (Chan et. al., 1995; Cook et al., 2009). To encounter these bactericidal components of the host, *Mtb* produces a number of scavenging enzymes for reactive oxygen and nitrogen species, such as superoxide dismutase, catalases, peroxidases (Manca et al., 1999; Voscuil et al., 2011), and truncated hemoglobins (Ouellet et al., 2002; Pathania et al., 2002). Together with antioxidants like thioredoxins, peroxiredoxins, and mycothiol, these enzymes provide highly efficient machinery to *Mtb* for the detoxification of toxic reactive species and balancing the redox environment of the cell (Bryk et al., 2000; Arner and Holmgren, 2000; Kumar et al., 2011; Zeida et al., 2015). Mycothiol, a functional analogue of glutathione, is the major low-molecular-mass thiol of actinomycetes and is present in high levels in *Mtb* (Newton et al., 1996; Reyes et al., 2018) where it acts as a major detoxification system not only against reactive oxygen and nitrogen species but also against toxins, electrophiles, and other reactive species (Buchmeier et al., 2003; Ung and Av-Gay, 2006). Under oxidative stress, mycothiol is oxidized into mycothione, which gets reduced and recycled into mycothiol by a flavoprotein, mycothione reductase, using NADPH as a co-factor (Patel and Blanchard, 1998; Patel and Blanchard, 1999). The genome of *Mtb* encodes a single copy of the *trx*R gene, encoding thioredoxin reductase (Bryk et al., 2002) and a NADPH-dependent disulfide reductase (Mtr), specific for mycothione (Patel and Blanchard, 1998). These flavoenzymes constitute crucial components of the antioxidant network of *Mtb* and play an important role in balancing the redox environment of the cell under different physiological conditions.

*Mtb* produces a unique hexa-coordinated flavohemoglobin (*Mtb*FHb) having antioxidant properties (Gupta et al., 2012). It belongs to a new class of two domain Hbs (type II FHbs) that is distinct from conventional type I FHbs (Bonamore and Boffi, 2008) and exclusively present in actinomycetes (Gupta et al., 2011). *Mtb*FHb carries a FAD-binding motif, similar to D-lactate dehydrogenases and oxidizes D-lactate into pyruvate in a FAD-dependent manner. The functional relevance of using D-lactate as an electron donor and the molecular mechanism by which *Mtb*FHb operates under oxidative stress are not known at present. The D-lactate metabolizing enzyme, D-lactate dehydrogenase (D-LDH), is missing in many mycobacterial species (Pinchuk et al., 2009), and a D-LDH activity has not been demonstrated in *Mtb* so far. It led to the speculation that *Mtb*FHb provides a unique ability to *Mtb* for eliminating the accumulation of D-lactate that is produced in the cells as a by-product of lipid peroxidation under oxidative stress. *Mtb*FHb represents the first example of a flavin and heme containing two-domain protein that mediates electron transfer from D-lactate to the heme domain *via* FAD bound to the reductase domain. Interestingly, the FAD binding site, similar to D-LDH, is present in type II FHbs of only few virulent mycobacteria. This co-factor binding site is absent or mutated in most of the pathogenic and non-pathogenic mycobacteria, suggesting that D-lactate-mediated electron transfer may not be the primary function of type II FHbs and may be specifically required in some pathogenic mycobacteria. The functional relevance of D-lactate oxidation and its role in modulating the physiological function(s) of *Mtb*FHb remain to be elucidated.

Here, we have identified a new co-factor binding site within the reductase domain of *Mtb*FHb, which is similar to the FAD-binding site of thioredoxin reductases (Dym and Eisenberg, 2001). Interestingly, this FAD-binding site overlaps with the previously identified D-LDH-type FAD-binding site of *Mtb*FHb, indicating that these FAD-binding sites may have cooperating functions in electron transfer. The present study unravels the functional relevance of two overlapping FAD-binding sites in *Mtb*FHb and provides new insights into the molecular mechanism of *Mtb*FHb function in *Mtb*. Here, we have demonstrated that *Mtb*FHb carries two distinct disulfide reductase activities and acts as a NADPH-dependent disulfide reductase and a D-lactate-dependent mycothiol disulfide reductase.

## Materials and Methods

### Bacterial Strains, Plasmids, and Culture Conditions

*Escherichia coli* JM109 and *E. coli* BL21DE3 were used routinely for the cloning and the expression of recombinant proteins, respectively. Cultures of *E. coli* were grown in Luria-Bertani (LB) or Terrific broth (containing 24 g of yeast extract, 12 g of Bacto-tryptone, 12.3 g of K_2_HPO_4_, and 2.3 g of KH_2_PO_4_) at 37°C and 180 rpm. Mycobacterial strains, *M. tuberculosis* H37Ra (designated as *Mtb*) and *M. smegmatis* mc^2^ 155, were used for the experimental studies and were grown in Middlebrook 7H10 agar (Difco) or 7H9 broth, supplemented with OADC (10% bovine serum albumin fraction V, dextrose, and sodium chloride), 0.2% glycerol, and 0.05% Tween 80. When required, kanamycin (Sigma) and hygromycin B (Sigma) were added at a concentration of 50 and 200 µg/ml, respectively, for *E. coli* and 25 and 50 µg/ml, respectively for selective growth of mycobacteria. The plasmids, p19Kpro (Garbe et al., 1994) and pVV16 (Stover et al., 1991), were used for the expression of recombinant genes in mycobacteria.

### Cloning, Expression, and Purification of Mycobacterial FHbs

Mycobacterial genes, Rv0385 and MSMEG_0719, encoding the *fhb* gene of *Mtb* and *fhbII* gene of *M. smegmatis*, respectively, were cloned and expressed as described earlier (Gupta et al., 2012; Thakur et al., 2018). Recombinant FHbs when expressed in *E. coli* appeared to be associated with the cell membrane and only a minor fraction of the protein came in soluble cytoplasmic fraction. The soluble protein fraction of *Mtb*FHb and *Ms*FHbII were purified essentially as described earlier (Gupta et al., 2012; Thakur et al., 2018). The protein and hemoglobin profiles were monitored at 280 and 414 nm, respectively. The *fhb gene* of *Mtb* and the *fhbII* gene of *M. smegmatis* were cloned on *E. coli*-mycobacterial expression vectors, p19Kpro, at *Bam*HI-*Hind*III sites and at *Nde*I-*Bam*HI sites on pVV16. Resulting plasmids, designated as p19kpro^MtbFHb^ and pVV16^MtbFHb^, were transformed *via* electroporation into *M. smegmatis* following standard procedure for the expression of recombinant proteins.

### Site-Directed Mutagenesis of *Mtb*FHb

Three cysteine residues (cys^188^, cys^289^, and cys^360^) of *Mtb*FHb were mutated individually using overlapping extension PCR to create three cysteine mutants, *Mtb*FHb^cys188ala^, *Mtb*FHb^cys289ala^, and *Mtb*FHb^cys360ala^, where the cysteine residue was mutated to alanine. A list of primers to create these mutants is provided in the **Supplementary Materials**. These mutants were cloned on an expression vector, pET9b, as described earlier for wild-type *Mtb*FHb (Gupta et al., 2012). Mutant *Mtb*FHb^cys188ala^ was expressed in *E. coli* and protein was purified as mentioned previously (Gupta et al., 2012). No protein expression was observed for two other cysteine mutants, *Mtb*FHb^cys289ala^ and *Mtb*FHb^cys360ala^.

### Treatment of *Mtb*FHb by Iodoacetamide

Iodoacetamide (IMA), a sulfhydryl-reactive alkylating agent, has been used to alkylate cysteines in *Mtb*FHb to check the role of cysteine in the protein function. Alkylation of the protein was carried out using a reduction alkylation system (G-biosciences). The reaction was set up in 500 µl of Tris-Cl (pH 7.2) buffer carrying 400 µg *Mtb*FHb mutant (*Mtb*FHb^cys188ala^), 2 µl of reduction buffer, and 10 µl of reductant solution from the kit. The reaction mixture was incubated at 37°C for 30 min to reduce the protein. Thereafter, alkylation of the protein was performed by adding 2.5 µl of alkylation buffer and 25 µl of 0.4 M of freshly prepared iodoacetamide. The reaction was incubated at room temperature for 30 min protected from light. Reaction mixture was then passed through G-50 desalting column to remove salts from the protein.

### Disulfide Reductase Activity Assay

Disulfide reductase activity of the protein was determined by reduction of insulin. Insulin stock solution (10 mg/ml) was prepared in 0.05 M Tris-Cl (pH 8.0) adjusting pH 2 to 3 by the addition of 1.0 M HCl and rapidly titrating the solution back to 8.0 with 1.0 M NaOH. The solution of insulin was perfectly clear and was stored at −20°C. The final reaction mixture (500 µl) contained 0.1 M potassium phosphate (pH 7.0), 2 mM EDTA, 0.13 mM insulin, and 0.33 mM dithiothreitol (DTT). The reaction was initiated by adding 2 µM protein and an increase in the absorbance at 650 nm was monitored to check the reduction of insulin. NADPH-dependent disulfide reductase activity of FHbs was checked by the reduction of DTNB [5,5’-dithiobis-(2-nitrobenzoic acid)] using NADPH as an electron donor. The assay was performed at room temperature in a buffer containing 100 mM potassium phosphate (pH 7.0), 2 mM EDTA, 5 mM DTNB, 250 µM NADPH, and 2 µM of FHb protein. Reduction of DTNB by *Mtb*FHb and *Ms*FHbII was monitored by the increase in absorbance at 412 nm. The kinetic constants were checked by varying the concentration of DTNB (50 µM to 500 µM) and keeping the concentration of protein constant (2 µM). DTNB reduction by both the FHbs was calculated by fitting the data to the Michaelis–Menten equation using GraphPad prism 8.

### Electron Transfer and Reduction of *Mtb*FHb Bound Co-Factors by NADPH and D-Lactate

Oxidation of NADPH and D-lactate as well as the reduction of FAD and the heme by *Mtb*FHb and *Ms*FHbII was checked spectrophotometrically as described previously (Singh et al., 2014). Briefly, the reaction was set up in 500 µl with 8 µM oxygenated *Mtb*FHb or *Ms*FHbII in 0.05 M potassium phosphate buffer (pH 7.2). Reaction was initiated after adding a specified amount of NADPH or D-lactate. Spectral changes exhibiting conversion of oxy-FHb into the ferrous form were monitored at different time intervals spectrophotometrically. Reduction of FAD was checked by the decrease in the absorbance at 460 nm, while reduction of heme was followed by the shift of Soret peak at 414 to 430 nm.

### NADP/NADPH Oxidase Activity

Oxidation of NADPH or NADH was checked spectrophotometrically by monitoring spectral changes at 340 nm. The assay was performed in 50 mM phosphate buffer (pH 7.0) containing 1 mM protein (*Mtb*FHb or *Ms*FHbII) and 1 mM EDTA. Reaction was initiated by adding 250 μM NADH or NADPH and spectra were recorded at 340 nm at specified time intervals as mentioned previously (Thakur et al., 2014).

### Circular Dichroism Spectroscopy

CD spectra of proteins were checked using a Jasco J-810 spectropolarimeter. Measurements in the far-UV region (250 to 190 nm) were carried out on protein solutions (0.4 mg/ml) employing a cell with a path length of 0.1 cm at 25°C. The mean residue ellipticity (θ) was calculated using a mean residue molecular mass of 110 Da. Each spectrum reported represents an average of 5 scans.

### Assay for Lipid Peroxidation

The lipid peroxides were determined through colorimetric assay using FOX II reagent (G-Bioscience) following manufacturer instructions. This reaction sequence involves the oxidation of ferrous to ferric ions by lipid peroxide with the subsequent binding of ferric ion to the ferric sensitive dye xylenol orange, yielding an orange to purple complex (color depends on the amount of –OOH present), which is measured at 560 nm. Lipid peroxidation in the cell sample was determined following the published procedure (Cha et al., 2004). Briefly, cell pellet (having 50 µg protein) was suspended in 1 ml of 50 mM Tris-Cl (pH 7.4) containing 0.5% SDS and sonicated, followed by removal of SDS after washing with deionized water twice. Resulting pellet was then dissolved in 1 ml methanol/chloroform (2:1 v/v) solution. The suspension was vortexed at room temperature. From this, 200 µl aliquot was taken and mixed with 800 µl of FOXII reagents and incubated for 30 min. The level of lipid peroxide in the cell was monitored by measuring the absorbance at 560 nm using the standard curve prepared from H_2_O_2_.

### Synthesis of Des-*Myo*-Inositol Disulfide Mycothione

Des-*myo*-inositol mycothione, a synthetic analog of mycothione, was synthesized chemically exactly following the published procedure (Patel and Blanchard, 1998). All experiments were performed at 25°C. ^1^NMR spectra were obtained at Jeol ECX300 MHz spectrophotometer. Details of synthesis are provided in **Supplementary Material**.

### Mass Spectrometric Analysis of Des-*Myo*-Inositol Mycothione

In the absence of any spectrophotometric method to detect D-lactate-mediated reduction of mycothiol disulfide (mycothione), we used mass spectrophotometric analysis of the product after reduction of des-*myo*-inositol disulfide mycothione. All assays were performed in a 100-µl reaction mixture containing 50 mM HEPES (pH 7.6) and 0.1 mM EDTA. *Mtb*FHb and *Ms*FHbII (5 µg each) were incubated individually with 100 μM des-*myo*-inositol mycothione along with 250 μM NADPH or 250 μM D-lactate at 25°C for 5 min and the product was analyzed *via* LC-MS using C18 column, with acetonitrile and water gradient. MSH was eluted at 3.81–4.20 min.

### Generation of *fhb* Gene Knock Out Mutants of Mycobacteria

To investigate the physiological function of mycobacterial FHbs, attempts were made to create FHb knock out strains of *Mtb* and *M. smegmatis* using plasmid vector pML 523 following the procedure described earlier (Song et al., 2009). Briefly, 1,100-bp upstream and downstream regions of the *fhb* gene of *Mtb* and *fhb*II gene of *M. smegmatis*, carrying 125-bp coding regions of amino terminus with *Spe*I restriction site at the N-terminus and *Swa*I restriction site at the C-terminus, were amplified by PCR (list of primers is provided in **Supplemental Materials**). Each amplified product was cloned at the *Spe*I and the *Swa*I sites of plasmid vector, pML523. Similarly, 1,100-bp downstream regions of the *fhb* gene of *Mtb* and the *fhb*II gene of *M. smegmatis* carrying 150-bp coding regions of the C-terminus was amplified individually carrying the *Pac*I restriction site at the N-terminus and the *Nsi*I restriction site at the C-terminus. These amplified products were cloned individually at the *Pac*I and *Nsi*I restriction sites of pML523 plasmid. Resulting gene knock out plasmids, pML523^MtbFHb^ carrying upstream and downstream regions of the *Mtb fhb* gene, and pML523^MsFHbII^ carrying upstream and downstream regions of *Msfhb*II gene were used transformed into *Mtb* and *M. smegmatis* for the *fhb* and *fhb*II gene deletion, respectively. Transformed cells were plated on Middlebrook 7H9 agar plates carrying 50 µg/ml hygromycin and transformants were screened for the expression of GFP (green fluorescent protein) and the presence of XylE after spraying 2% catechol using published procedure (Song et al., 2009). No colonies were obtained for *Mtb* after repeated attempts but few colonies were obtained for *M. smegmatis*. These colonies were screened for the gene knock out. Deletion of the *fhb*II gene in *M. smegmatis* was confirmed through multiple PCR primer sets specific for the gene and resulting strain was designated as *Ms*Δ*fhb*II (**Figure S7**).

### Quantitative RT-PCR

Wild type, *Ms*Δ*fhb*II, and *Mtb fhb* gene overexpressing cells of *M. smegmatis* were cultured in Middlebrook 7H9 medium at 37°C at 200 rpm for 18 h. Thereafter, cells were taken out and centrifuged at 14,000 rpm. RNA was purified using TRI-Reagent (Sigma-Aldrich) as per manufacturer instructions. All the samples were treated with DNA-free DNase (Ambion) to remove any contaminating DNA. Thereafter, cDNA was synthesized using the Superscript III first strand synthesis system and qRT-PCR was performed in a Bio-Rad cycler using 2x SYBR real-time PCR pre-mix (Sso Fast™ Evagreen Supermix, Bio-Rad). Primers used for qRT-PCR are listed in **Supplementary Material**. The *sig*A gene was used as housekeeping control. Relative fold change in the transcript level of the *fhb*II gene and *Mtb fhb* gene in *M. smegmatis* was calculated. All qRT-PCR experiments were performed with three replicates and signals were normalized to the housekeeping *sig*A mRNA and quantified by the Livak (2^−ΔΔCt^) method (Livak and Schmittgen, 2001). Statistical analysis was performed using one-tailed *t*-test, and the results were plotted based on average and standard deviation taken from three replicates using GraphPad Prism version 8.4.3 for windows.

### Growth Properties and Stress Responses of *Ms*Δ*fhb*II and Wild-Type *M. smegmatis* After Expression of the *Mtb fhb* Gene

Since the *Ms*Δ*fhb*II strain of *M. smegmatis* is resistant to hygromycin, we transformed pVV^MtbFHb^ into wild type and *Ms*Δ*fhb*II strains of *M. smegmatis* to select kanamycin-resistant colonies expressing the *Mtb fhb* gene. Growth properties of recombinant strains along with its isogenic wild-type *M. smegmatis* were checked under aerobic and oxidative stress conditions. Overnight grown cells of wild type and *Mtb*FHb expressing *Ms*Δ*fhb*II strains of *M. smegmatis* as well as their isogenic control cells were freshly inoculated into Middlebrook 7H9 growth medium to adjust the final cell OD_600_ at 0.05 nm. Hygromycin (25 µg/ml) or kanamycin (25 µg/ml) was supplemented wherever required. Growth profiles of these cells were checked periodically by taking optical density at 600 nm. H_2_O_2_, CHP, and diamide were used as oxidants to impose oxidative stress. For CFU count, *Mtb* cells were grown to mid-log phase and washed twice in 7H9 medium and single-cell suspension was then prepared by centrifuging the culture at 8,000 rpm for 10 min in the presence of sterilized glass beads. The OD of cells was adjusted to 0.03. Cells were then exposed to oxidants for 30 min and CFU was checked after plating and counting colonies after 2 days.

### Statistical Analysis

Statistical analysis of the data was carried out using two-way ANOVA followed with a post-hoc Dunnett multiple comparison test and one-tailed *t*-test. Transcript level was quantified by the Livak (2^−ΔΔCt^) method, and results were plotted based on average and standard deviation taken from three replicates using GraphPad Prism version 8.4.3 for windows.

## Results

### *Mtb*FHb Carries Two Overlapping FAD-Binding Sites

**Figure 1:**
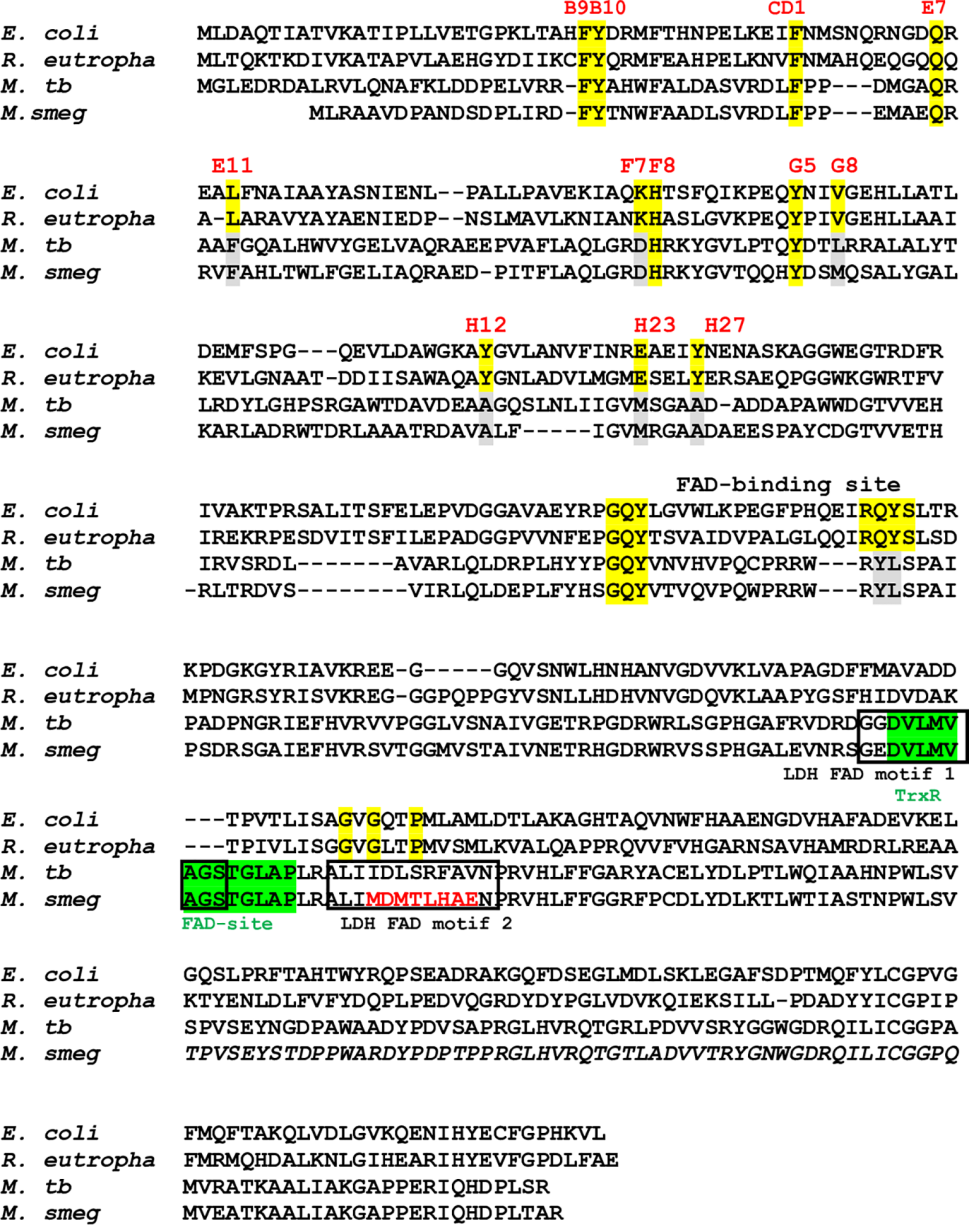
Sequence alignment of type I FHbs of *E. coli* and *R. eutropha* with type II FHbs of *Mtb* and *M. smegmatis* showing key residues of globin and reductase domains. The conserved residues within the globin domain are marked and highlighted in yellow and mutated residues of type II residues are shown in gray. Putative thioredoxin reductase-type FAD binding site is highlighted in green [D(x) 8GxxP] and LDH-type FAD binding sites 1 and 2 [A(x)7AxN, G(x)7 GS] are shown in black boxes. Mutation within the intergenic region of D-LDH binding site of *Ms*FHbII is shown in red. Bacterial strain are designated as *E. coli* (*Escherichia coli*), *R. eutropha* (*Ralstonia eutropha*), *M. tb* (*Mycobacterium tuberculosis*), and *M. smeg* (*Mycobacterium smegmatis*).

*Mtb*FHb, encoded by Rv0385 gene of *Mtb*, has been identified as a novel hexa-coordinated flavo-heme protein carrying a globin and a reductase domain (Gupta et al., 2012). The reductase domain of *Mtb*FHb has a FAD-binding motif similar to respiratory D-LDH and oxidizes D-lactate into pyruvate in a FAD-dependent manner. FAD-binding to this co-factor binding motif of *Mtb*FHb has been validated earlier by site-directed mutagenesis (Gupta et al., 2012). Structure-based sequence alignment of *Mtb*FHb with its homologs (type II FHbs) in mycobacteria revealed that except *Mtb* and few virulent mycobacteria, the D-LDH type FAD-binding site is absent or mutated in other type II FHbs of mycobacteria and other actinomycetes (**Figure S1**), indicating that the oxidation of D-lactate may not be the primary function of *Mtb*FHb and its homologs. Detailed sequence analysis revealed the presence of a new FAD-binding site (D(x)8GxxP) within the reductase domain of *Mtb*FHb, which is similar to the FAD-binding site of thioredoxin reductases (Dym and Eisenberg, 2001). This FAD-binding motif overlaps with previously identified D-LDH-type FAD-binding site in *Mtb*FHb and appeared conserved among homologs of *Mtb*FHb present in mycobacteria and other actinomycetes (**Figures 1** and **S1**), suggesting that the primary function of *Mtb*FHb and its homologs may be related to disulfide oxidoreductases.

### *Mtb*FHb Acts as a Disulfide Reductase

**Figure 2:**
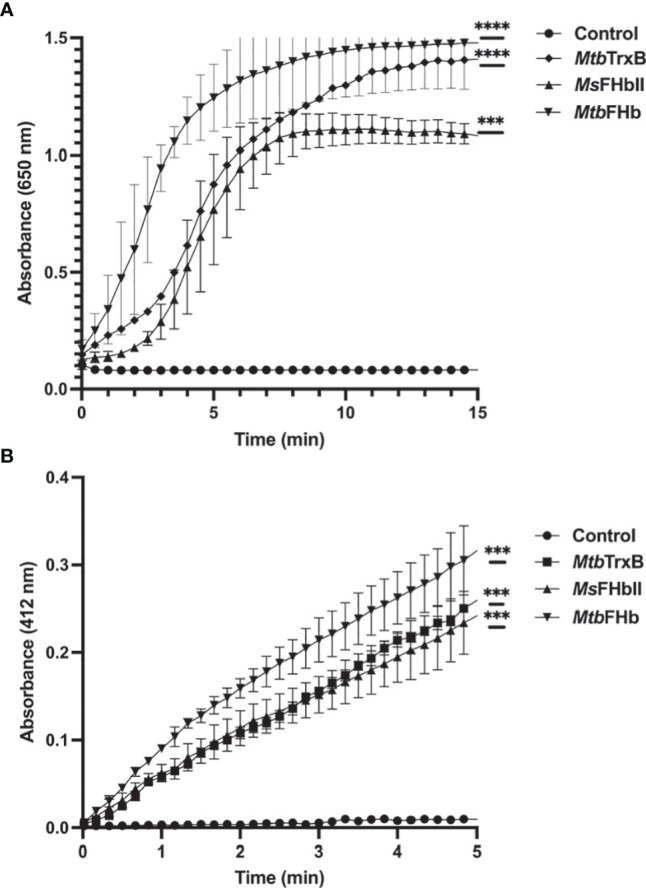
**(A)** Insulin disulfide reductase activity of *Mtb*FHb, *Ms*FHbII, and trxB of *M. tuberculosis*. Insulin reduction assay was set up in 500 µl containing 0.1 M potassium phosphate (pH 7.0), 2 mM EDTA, 0.13 mM insulin, and 0.33 mM DTT. The reaction was carried out at 25°C by adding 2 µM proteins (*Mtb* FHb, *Ms* FHbII, or *Mtb* trxB) and monitoring insulin reduction by the increase in optical density at 650 nm. **(B)** NADPH-dependent reduction of DTNB by *Mtb*FHb, *Ms*FHbII, and trxB. The assay was carried out at 25°C in a 500-µl reaction mixture containing 100 mM potassium phosphate (pH 7.0), 2 mM EDTA, 5 mM DTNB, and 250 µM NADPH. Reaction was carried out by adding 2 µM proteins (*Mtb*FHb, *Ms*FHbII, or *Mtb* trxB). The control reaction was set up without any protein. Reduction of DTNB was monitored by the increase in absorbance at 412 nm. Experiments were repeated at least five times. Results are presented as the mean values ± standard deviations. Two-way ANOVA was employed to analyze statistical significance. ^***^ *p* < 0.001, ^****^ *p* < 0.0001.

Since thioredoxin reductase-type FAD-binding motif in *Mtb*FHb and other type II FHbs are fully conserved, we attempted to see if *Mtb*FHb and its homologs are in fact functioning as novel heme containing disulfide reductase. To validate that we selected type II FHb (*Ms*FHbII) of *M. smegmatis*, which has close sequence similarity with *Mtb*FHb and carries similar thioredoxin reductase-like FAD-binding motif (**Figure 1**), we first tested whether *Mtb*FHb and *Ms*FHbII are able to reduce insulin, similar to thioredoxin reductases. Protein disulfide reductase activity of these FHbs, taking thioredoxin reductase (trxB) of *Mtb* as a reference, was tested by insulin disulfide reduction assay where the protein catalyzes the reduction of insulin in the presence of thiol reductant, dithiothreitol (DTT). Both *Mtb*FHb and *Ms*FHbII displayed rapid reduction of insulin in the presence of DTT (**Figure 2A**), very similar to trxB, indicating that *Mtb*FHb and *Ms*FHbII are acting as a disulfide reductase and their thioredoxin-type FAD binding site is functional. Earlier, we created an *Mtb*FHb mutant, carrying mutations at GS residues of the conserved D-LDH type FAD binding motifs (GX7GS.AX7AXN). This mutant appeared defective in FAD binding (Gupta et al., 2012) and oxidation of D-lactate into pyruvate. We found that this mutant is unable to oxidize NADPH also unlike wild-type *Mtb*FHb. Since GS residues of D-LDH-type FAD-binding motif also overlaps with thioredoxin reductase-type FAD-binding site of *Mtb*FHb (**Figure 1**), it is quite likely that a change in the overlapping region of two FAD-binding sites has disrupted the association of *Mtb*FHb with FAD at both of these locations.

### Disulfide Reductase Activity of *Mtb*FHb Is NADPH Dependent

*Mtb*FHb and *Ms*FHbII lack a NADH binding site and their NADH oxidase activity is attenuated (Gupta et al., 2012; Thakur et al., 2018) unlike conventional type I FHb (*Ms*FHbI) of *M. smegmatis* (Thakur et al., 2014) and other bacteria. Since thioredoxin reductases are NADPH-dependent oxidoreductases and *Mtb*FHb and *Ms*FHbII carry thioredoxin reductase-like FAD binding motif, we checked whether these FHbs are able to oxidize NADPH. The oxidation of NADPH appeared quite fast in the presence of *Mtb*FHb and *Ms*FHbII, indicating that these FHbs are using NADPH as an electron donor instead of NADH (**Figure S2**). We further checked disulfide reductase activities of *Mtb*FHb and *Ms*FHbII in the presence of a generic disulfide substrate, DTNB (5,5’-dithiobis,2-nitrobenzoic acid), using NADH, NADPH, and D-lactate to check the involvement of these electron donors in this process. Reduction of DTNB by both the proteins increased in the presence of NADPH in a time-dependent manner, similar to the trxB of *Mtb* (**Figure 2B**), but D-lactate and NADH were not utilized as electron donors for the DTNB reduction. NADPH-dependent reduction of DTNB by *Mtb*FHb and *Ms*FHbII appeared comparable to that of trxB of *Mtb*. These results demonstrated that *Mtb*FHb and *Ms*FHbII carry a NADPH-dependent disulfide reductase activity. *Mtb*FHb and *Ms*FHbII reduced DTNB with *K*_m_ values of 98.5 ± 6.8 μM and 93.7 ± 7.6 μM, respectively, thereby indicating that the specificity of these FHbs is significantly higher towards DTNB as compared to Mtr of *Mtb*, which has 3300 ± 700 μM *K*_m_ for the reduction of DTNB (Patel and Blanchard, 1999). The DTNB reduction by *Mtb*FHb and *Ms*FHbII was estimated as 1608 ± 66 mM/min/mg and 1079 ± 48 mM/min/mg, respectively.

### Role of Cysteines in Disulfide Reductase Activity of *Mtb*FHb

**Figure 3:**
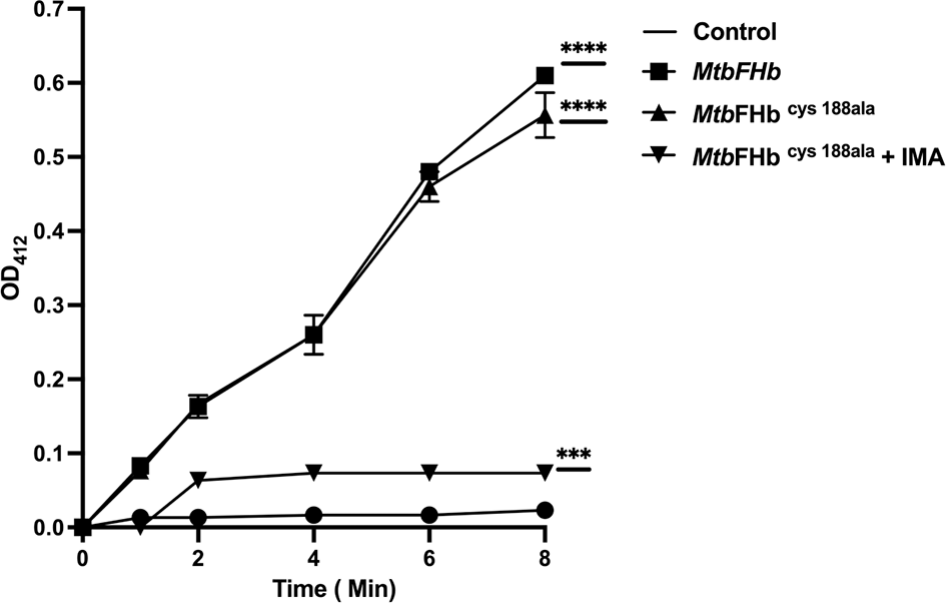
Reduction of DTNB by *Mtb*FHb and mutant *Mtb*FHb^cys188ala^. Iodoacetamide (IMA) treatment of *Mtb*FHb^cys188ala^ mutant protein was carried out first in 500 µl of Tris-Cl (pH 7.2) buffer carrying 400 µg of protein, 2 µl of reduction buffer, and 10 µl of reductant solution from the kit (G-bioscience). The reaction mixture was incubated at 37°C for 30 min to reduce the protein. Thereafter, alkylation of the protein was performed by adding 2.5 µl of alkylation buffer and 25 µl of 0. 4 M of freshly prepared IMA. The reaction was incubated at room temperature for 30 min protected from light. Reaction mixture was then passed through G-50 desalting column to remove salts from the protein. DTNB reduction assay mixture contained wild type and the mutant *Mtb*FHb (with 50 micromole heme content), 0.5 mM NADPH, and 0.1 mM DTNB. *Mtb*FHb^cys188ala^ (IMA) is designated for *Mtb*FHb^cys188ala^ treated with IMA. The change in absorbance at 412 nm was monitored at 30°C at a specified time. Data are shown as mean ± standard deviation of five independent scans. ***p < 0.001, ****p < 0.0001.

Although the reductase domain of *Mtb*FHb carries three cysteines, it lacks a typical disulfide active site (CXXC) unlike conventional disulfide reductases. To investigate the role of cysteines in protein function, site-directed mutagenesis has been carried out to create three cysteine mutants of *Mtb*FHb (*Mtb*FHb^cys188ala^, *Mtb*FHb^cys289ala^, and *Mtb*FHb^cys360ala^) where cys^188^, cys^289^, and cys^360^ residues were mutated individually into alanine. Mutation in cys^188^ of *Mtb*FHb did not affect the protein function of *Mtb*FHb as *Mtb*FHb^cys188ala^ mutant exhibited NADPH-dependent reduction of DTNB appeared very similar to wild type (**Figure 3**). The other two *Mtb*FHb mutants, *Mtb*FHb^cys289ala^ and *Mtb*FHb^cys360ala^, could not be overexpressed in *E. coli*, possibly due to rapid protein degradation. It is likely that these cysteine residues are crucial for conformational stability and functional activity of the protein as these are conserved among all type II FHbs of mycobacteria. To overcome this problem, we selected cysteine mutant *Mtb*FHb^cys188ala^ that carries only two cysteines and subjected it to IMA treatment after reduction to block the formation of disulfide bond if that is created within two cysteine (cys^289^ and cys^360^) residues of the protein. NADPH-dependent reduction of DTNB by *Mtb*FHb^cys188ala^ was reduced significantly after IMA treatment as compared to the untreated one. These results suggested that cys^289^ and cys^360^ residues are involved in modulating the disulfide reductase activity of *Mtb*FHb.

### Electron-Transfer During Oxidation of NADPH and D-Lactate by *Mtb*FHb

**Figure 4:**
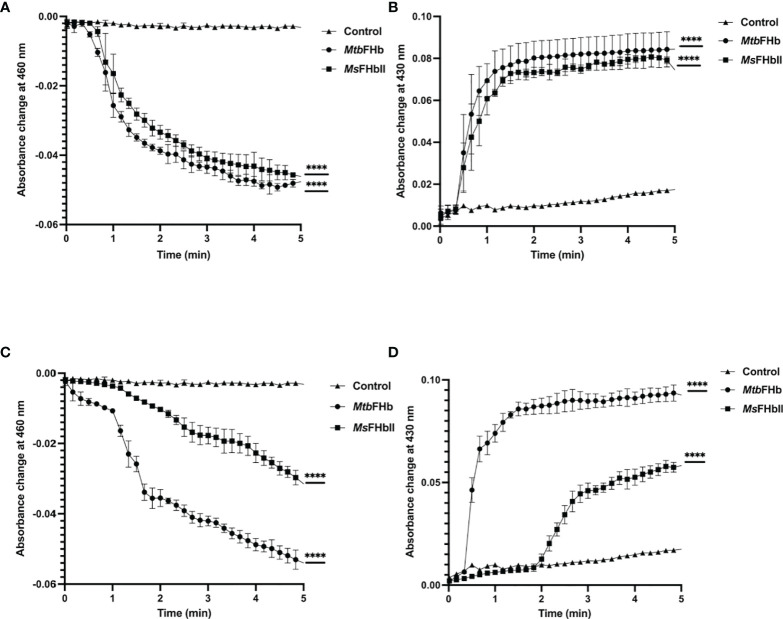
Profile of heme and FAD reduction during oxidation of NADPH and D-lactate by *Mtb*FHb and *Ms*FHbII. Reaction was set up in 500 µl with 8 µM oxygen bound *Mtb*FHb or *Ms*FHbII in 0.05 M potassium phosphate buffer (pH 7.2). Reaction was initiated after adding 250 μM D-lactate or 250 μM NADPH and recording the spectral changes with change in time. Heme reduction was followed by the shift in Soret peak from 414 nm to 430 nm **(B, D)**, while reduction of FAD was checked by the decrease in absorbance at 460 nm **(A, C)**. Experiments were repeated at least five times and data are shown as mean ± standard deviation of five independent scans. Two-way ANOVA was employed to analyze statistical significance. ^****^ *p* < 0.0001.

To understand the mechanism by which *Mtb*FHb participates in electron transfer using NADPH and D-lactate as electron donors, we checked the pattern of heme and FAD reduction during oxidation of NADPH and D-lactate by *Mtb*FHb and compared it with *Ms*FHbII that carries a D-LDH type FAD-binding motif with mutated intergenic region (**Figure 1**). The electron transfer during oxidation of D-lactate and NADPH occurs in a two-step process, wherein these electron donors donate electrons and reduce the FAD, which further transmits electrons to the heme iron. Therefore, we compared the efficiency of redox state changes in the FAD and the heme moieties in *Mtb*FHb and *Ms*FHbII and pattern of electron transfer during oxidation of D-lactate and NADPH. When *Mtb*FHb was reduced with NADPH, a rapid decrease occurred at 460 nm and the optical spectrum of the FAD changed very fast at 250 µM NADPH, reaching to fully reduced state within 2 min and simultaneously displaying a fast increase at 430 nm, which indicated rapid reduction of the heme iron. Similar behavior was observed in the case of *Ms*FHbII (**Figures 4A, B**). These data demonstrated that those two reductive steps, which include reduction of FAD by NADPH followed by reduction of heme by an intra-molecular electron transfer, occur more or less in a similar manner in the case of *Mtb*FHb and *Ms*FHbII. However, when D-lactate was used as an electron donor, the optical spectrum of the flavin at 460 nm changed very fast in the presence of *Mtb*FHb and reached to fully reduced state in the presence of 250 µM D-lactate, whereas reduction of FAD by D-lactate occurred very slowly and got reduced only partially in the case of *Ms*FHbII under identical conditions (**Figure 4C**). Similarly, the rate of heme reduction by D-lactate appeared nearly 4- to 5-fold faster in *Mtb*FHb in comparison to *Ms*FHbII (**Figure 4D**), indicating that the transfer of electrons to the FAD *via* D-lactate is not very efficient in the case of *Ms*FHbII in comparison to *Mtb*FHb. Thus, both FHbs appear to have similar NADPH-dependent disulfide reductase activities, but the efficiency of electron-transfer by D-lactate and reduction of FAD and the heme iron appeared extremely slow in *Ms*FHbII in comparison to *Mtb*FHb. These results suggested that the D-lactate and NADPH are utilized efficiently as electron donors by *Mtb*FHb and both its FAD-binding sites are efficient in electron transfer, unlike *Ms*FHbII, where transfer of electrons occurs very slowly in the presence of D-lactate.

### D-Lactate Inhibits Oxidation of NADPH and FAD-Mediated Electron Transfer by *Mtb*FHb

**Figure 5:**
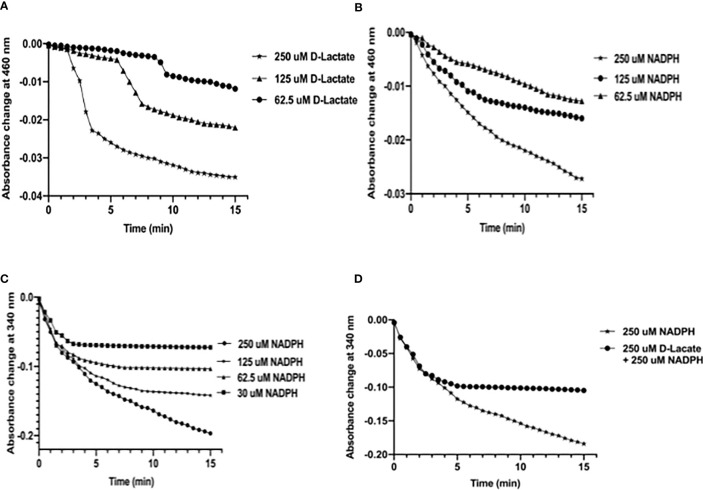
D-lactate inhibits oxidation of NADPH by *Mtb*FHb. **(A)** Profile of FAD reduction by *Mtb*FHb in the presence of different concentrations of D-lactate. **(B)** Profile of FAD reduction by *Mtb*FHb in the presence of varying concentrations of NADPH. **(C)** Oxidation of NADPH by *Mtb*FHb. **(D)** Inhibition of NADPH oxidation by *Mtb*FHb in the presence of D-lactate. The reaction was set in 500 µl containing specified amount of NADPH or D-lactate in 0.5 M potassium phosphate buffer (pH 7.2) and 1 mM EDTA. Baseline level of FAD reduction or NADPH oxidation was set before addition of the protein in the reaction mixture. Reduction of heme in the presence of different concentrations of D-lactate or NADPH was monitored spectrophotometrically at 460 nm after adding *Mtb*FHb (8 µM) in the reaction mixture. Data are shown as mean ± standard deviation of three independent experiments. Two-way ANOVA was employed to analyze statistical significance. ^****^ *p* < 0.0001.

Since two overlapping FAD-binding sites of *Mtb*FHb appeared fully functional in electron transfer, we checked how these two co-factors’ binding sites coordinate their function. Our earlier studies demonstrated that FAD associates with *Mtb*FHb in 1:1 stoichiometry (Gupta et. al., 2012) and one FAD moiety remains attached with one molecule of *Mtb*FHb; thus, only one FAD-binding site may be operative at a time with respect to its specific electron donor (NADPH or D-lactate). It led us to explore how these two overlapping FAD-binding sites in *Mtb*FHb operate and modulate protein function in the presence of two different electron donors (NADPH and D-lactate). Therefore, we checked the reduction of FAD by NADPH alone and in combination with D-lactate by monitoring spectral changes at 460 nm and 340 nm to check the reduction of FAD and the oxidation of NADPH, respectively. The reduction of FAD occurs very fast and gets fully saturated at 250 µM when D-lactate and NADPH are used as electron donor individually, whereas at lower concentrations, it remains partially reduced (**Figures 5A, B**). It was further confirmed by the oxidation of NADPH by *Mtb*FHb. (**Figure 5C**), which indicated that *Mtb*FHb oxidizes NADPH very efficiently in a concentration-dependent manner (**Figure 5C**). As shown in **Figure 5A**, FAD bound with *Mtb*FHb gets fully reduced in the presence of 250 µM NADPH but the oxidation of NADPH was inhibited completely when D-lactate was incorporated in the reaction (**Figure 5D**). These results suggested that electron transfer by NADPH to the FAD in *Mtb*FHb gets restricted in the presence of D-lactate.

### D-Lactate Mediates Conformational Changes in *Mtb*FHb

**Figure 6:**
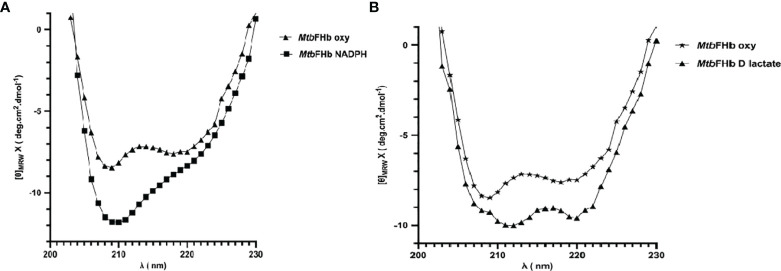
Secondary structure analysis of *Mtb*FHb. The near-UV CD spectra of native *MTb*FHb after reduction with NADPH **(A)** and D-lactate **(B)**. Protein sample (20 µM) was prepared in 50 mM Tris-Cl (pH 7.8). Spectra were recorded individually in the far-UV region (250 to 190 nm) and were carried out on protein solutions (0.4 mg/ml) employing a cell with a path length of 0.1 cm at 25°C after addition of NADPH (10 µM) or D-lactate (10 µM). Each spectrum reported represents an average of 5 scans.

The addition of D-lactate to the ferric *Mtb*FHb (with Soret and visible bands at 414 and 535/570 nm) results in a species with Soret and visible bands at 426 and 529/558 nm, indicating reduction of the heme iron (**Figure S3**). Since D-lactate inhibited oxidation of NADPH by *Mtb*FHb, it is quite likely that a conformational change takes place in *Mtb*FHb in the presence of D-lactate. To test this possibility, we checked far-UV-CD spectra of the *Mtb*FHb in the presence and the absence of D-lactate to see if D-lactate induces any structural change in *Mtb*FHb that prevents access of NADPH towards the FAD moiety and disrupts the electron transfer. The CD spectrum of native *Mtb*FHb exhibited two negative peaks at 208 and 222 nm (**Figure 6A**) that are characteristics of alpha-helical structure, as reported for trHbN of *Mtb* (Lama et al., 2006). In the presence of D-lactate, the negative peak at 222 nm gets more pronounced and a new peak appears at 212 nm, which indicated significant conformational change in the heme domain. No such change has been observed in *Mtb*FHb in the presence of NADPH (**Figure 6B**). These results confirmed a conformational change in *Mtb*FHb during its interaction with D-lactate.

### D-Lactate Changes the Specificity of *Mtb*FHb Towards Mycothiol Disulfide

**Figure 7:**
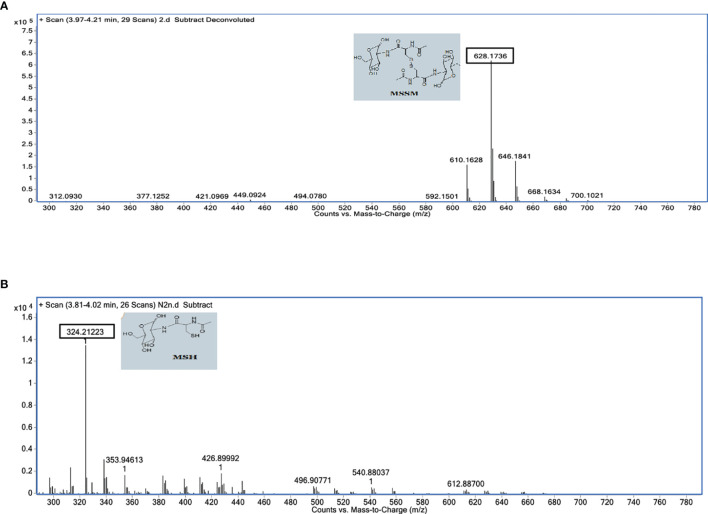
Mass spectrophotometric analysis of des-*myo*-inositol mycothiol (synthetic analog of mycothione) reduction in the absence **(A)** and the presence **(B)** of D-lactate. Assays were performed in a 100-µl reaction mixture containing 50 mM HEPES (pH 7.6) and 0.1 mM EDTA. *Mtb*FHb and *Ms*FHbII (5 µg each) were incubated individually with 100 μM des-*myo*-inositol mycothione along with 250 μM NADPH or 250 μM D-lactate at 25°C for 5 min and the product was analyzed *via* LC-MS using C18 column, with acetonitrile and water gradient. MSH was eluted at 3.81–4.20 min. MSH, *Myo*-inositol mycothiol; MSSH, des-*myo*-inositol mycothiol.

In an attempt to understand the molecular mechanism by which disulfide reductase activity of *Mtb*FHb is modulated by two different co-factors, we checked how it would respond towards mycothiol that exists as a major low-molecular-mass thiol in mycobacteria (Newton et al., 1996) and tested whether *Mtb*FHb is able to use mycothiol disulfide as a substrate that gets accumulated under oxidative stress. Mycothiol is structurally unusual, containing an alpha 1-1 glycosidic linkage between the two monosaccharides, D-glucosamine and myo-inositol. A structural analogue of oxidized mycothiol, des-*myo*-inositol mycothiol, has been successfully used previously to test mycothione reductase activity of a NADPH-dependent mycothione reductase (Patel and Blanchard, 1998; Patel and Blanchard, 1999). Therefore, we synthesized this synthetic analogue of mycothione (**Figure S4**) using the published procedure (see **Supplementary Information**) and used it as a substrate to check whether *Mtb*FHb displays any mycothione reductase activity using NADPH or D-lactate as an electron donor. Due to difficulty in checking the oxidation of D-lactate spectrophotometrically, we used a mass spectrometry-based approach to check the reduction of des-*myo*-inositol mycothiol. *Mtb*FHb was incubated with D-lactate and NADPH separately and the reaction products were analyzed *via* LC-MS to confirm reduction of disulfide linkage of the product. When NADPH was used as an electron donor, no visible reactivity was observed on des-*myo*-inositol mycothiol in the presence of *Mtb*FHb (**Figure S5**). Interestingly, when *Mtb*FHb was incubated with des-*myo*-inositol mycothiol in the presence of D-lactate, it exhibited the appearance of a main peak corresponding to half the size of the original substrate (**Figures 7A, B**), indicating reduction of disulfide linkage of the substrate. These results suggested that *Mtb*FHb is capable of reducing di-mycothiol in the presence of D-lactate but not in the presence of NADPH, indicating that the interaction of D-lactate with *Mtb*FHb mediates a conformational change that attenuates its interactions with NADPH and changes its substrate specificity towards mycothiol disulfide.

### Deletion of the *fhb* Gene in *Mtb* and *fhbII* Gene in *M. smegmatis*

**Figure 8:**
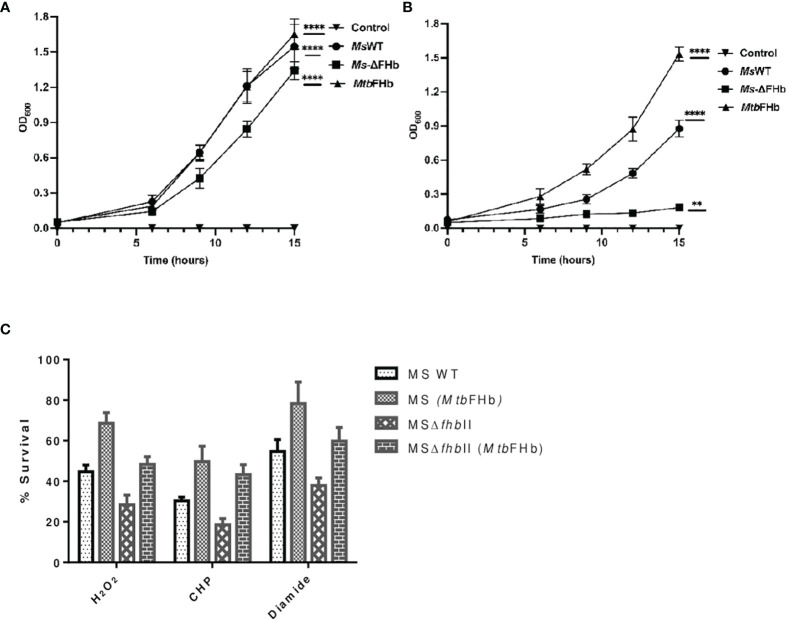
Implications of *Mtb fhb* gene expression on growth properties of MsΔ*fhb*II and wild-type strains of *M. smegmatis.* Growth profile of wild type and MsΔ*fhb*II strains of *M. smegmatis* overexpressing *Mtb fhb* gene **(A)** under aerobic and **(B)** under oxidative stress conditions in the presence of 20 mM CHP. **(C)** Survival of *Ms*Δ*fhb*II mutant of *M. smegmatis* after complementation of *Mtb fhb* gene during exposure to different oxidants, H_2_O_2_ (20 mM), CHP (10 mM), and diamide (10 mM). For CFU count, *Mtb* cells were grown to mid-log phase and washed twice in 7H9 medium and single-cell suspension was then prepared by centrifuging the culture at 8,000 rpm for 10 min in the presence of sterilized glass beads. The O.D. of cells was adjusted to 0.03. Cells were then exposed to oxidants for 30 min and CFU was checked after plating and counting colonies after 2 days. Data represent mean values with standard deviations (error bars) from three independent experiments. Data are shown as mean ± standard deviation of three independent experiments. Two-way ANOVA was employed to analyze statistical significance level with *p*< 0.05. MsWT: Wild-type *M. smegmatis*; Ms+*Mtb*FHb: *M. smegmatis* carrying *Mtb fhb* gene; MSΔ*fhb*II: *M. smegmatis* carrying deletion of *fhbI*I gene; MSΔ*fhb*II+MtbFHb: *Mtb fhb* gene complemented in MSΔ*fhb*II. **p < 0.01, ****p < 0.0001.

To assess the physiological function of *Mtb*FHb, we attempted to construct an *fhb* gene deleted strain of *Mtb*. Unfortunately, no viable colonies of *Mtb* were obtained despite our repeated attempts to knock out *fhb* gene. Since *Ms*FHbII shares close sequence similarity with *Mtb*FHb and exhibits similar NADPH-dependent disulfide reductase activity, we constructed a *fhb*II gene knock out strain of *M. smegmatis* and used it as a model to evaluate the physiological function of *Mtb*FHb. Disruption of the *fhb*II gene in *M. smegmatis* was confirmed through PCR analysis of the genome of wild type and the mutant strain (**Figure S6**). The selected mutant strain, thereafter, was designated as *Ms*Δ*fhb*II. The growth of *Ms*Δ*fhb*II appeared slightly slow under aerobic conditions in comparison to its wild-type counterpart (**Figure 8A**). However, when testing the response of *Ms*Δ*fhb*II under oxidative stress, it exhibited enhanced growth sensitivity towards oxidants, most prominently in the presence of cumene hydroperoxide (CHP), which inhibited the growth of *Ms*Δ*fhb*II completely at 20 mM, whereas wild-type *M. smegmatis* was able to resume its growth after a period of initial lag under similar conditions (**Figure 8B**).

### Expression of the *Mtb fhb gene* Promotes Tolerance Towards Different Oxidants and Reduces Lipid Peroxidation in *Ms*Δ*fhb*II and Wild-Type *M. smegmatis*

In an attempt to understand the physiological role of *Mtb*FHb, we complemented the *fhb* gene of *Mtb* in *Ms*ΔFHbII and also overexpressed it in wild-type *M. smegmatis.* Expression of the *Mtb fhb* gene in wild type and *Ms*Δ*fhb*II strains of *M. smegmatis* was checked through qRT-PCR, which indicated nearly 2.5- to 3-fold increase in the transcript level of the *Mtb fhb* gene with respect to the control (**Figure S7**). Also, no signal for *Msfhb*II gene was observed in the *Ms*Δ*fhb*II strain of *M. smegmatis.* As shown in **Figure 8C**, complementation of the *Mtb fhb* gene significantly increased the resistance of the *Ms*Δ*fhbII* strain towards different oxidants in comparison to its isogenic control cells. Moreover, overexpression of the *Mtb fhb* gene also enhanced the resistance of wild-type *M. smegmatis* towards various oxidants. The lipid content of mycobacterial cell wall is unusually high and its unsaturated fatty acids may get decomposed under oxidative stress; thus, it can be hypothesized that being a membrane-associated protein, *Mtb*FHb may protect the damage of lipids and the integrity of cell wall due to its antioxidant activity. Since D-lactate is produced as a by-product of lipid peroxidation and *Mtb*FHb is able to metabolize D-lactate, we assessed the connection of *Mtb*FHb with lipid peroxidation to understand its role under oxidative stress. Therefore, we checked the level of lipid peroxidation in the *Mtb fhb* gene expressing *Ms*Δ*fhb*II and wild-type *M. smegmatis* under oxidative stress along with their isogenic control cells. The level of lipid hydroperoxide appeared significantly higher in the *Ms*ΔFHbII strain as compared to wild-type *M. smegmatis* under oxidative stress (**Table 1**). When the *Mtb fhb* gene was over-expressed in these strains, the level of lipid hydroperoxide reduced to 25% to 30%.

**Table 1:** Cellular level of lipid hydroperoxide in *fhb* gene expressing cells of wild type and Δ *Ms*FHbII strains of *M. smegmatis*.

Sl. No	Mycobacterial strains	Cellular level of lipid peroxide nmol/mg protein
1	*M. smegmatis* (WT)	0.38 ± 0.03
2	*M. smegmatis* (WT)+H_2_O_2_	31.50 ± 3.55
3	*M. smegmatis* (Δ *Ms*FHbII)	1.17 ± 0.08
4	*M. smegmatis* (Δ *Ms*FHbII) + H_2_O_2_	45.35 ± 3.24
5	*M. smegmatis* (WT) + *Mtb*FHb	0.33 ± 0.15
6	*M. smegmatis* (WT) + *Mtb*FHb + H_2_O_2_	22.60 ± 3.54
7	*M. smegmatis* (Δ *Ms*FHbII) + *Mtb*FHb	0.38 ± 0.02
8	*M. smegmatis* (Δ *Ms*FHbII) + *Mtb*FHb + H_2_O_2_	32.92 ± 2.11

MtbFHb expressing cells of wild-type M. smegmatis and M. smegmatis (Δ MsFHbII) along with their isogenic control cells were grown in Middlebrook 7H9 broth for 12 h at 37°C. Cells were then treated with 20 mM H_2_O_2_ for 30 min and the level of lipid hydroperoxide was estimated as mentioned in Materials and Methods. Data represent the average of three replicates. Statistical analysis was performed with the Student’s t-test at a significance level of p < 0.05.

## Discussion

*Mtb* is armed with a number of antioxidant defense mechanisms and compensatory metabolic pathways that allow the pathogen to cope with hazardous intracellular environment where copious amounts of reactive oxygen and nitrogen species are produced *via* the host defense system. Thiol-disulfide oxidoreductases like thioredoxin reductase (trxB) and mycothiol disulfide reductase are vital components of the antioxidant defense system of *Mtb* that regulate the activity of many cellular proteins through reversible reduction of their disulfide bond and play a central role in redox homeostasis (Kumar et al., 2011; Trivedi et al., 2012). *Mtb*FHb sets the first example of a heme-containing oxidoreductase having two distinct disulfide reductase activities that may play a pivotal role in redox homeostasis and defense from toxic oxidants during the intracellular regime of *Mtb*.

Phylogenetically, *Mtb*FHb and its homologs form a separate cluster from conventional type I FHbs (Gupta et al., 2011). Our study demonstrated that *Mtb*FHb acts as a unique electron-transferring protein that utilizes NADPH and D-lactate as electron donors to carry out two different disulfide reductase activities. Conservation of thioredoxin reductase-like FAD-binding site in homologs of *Mtb*FHb suggested that these FHbs may be acting as novel heme containing disulfide-reducing enzymes. This is supported by the observation that *Mtb*FHb and its homolog in *M. smegmatis* (*Ms*FHbII) are able to carry out insulin and NADPH-dependent DTNB reduction very similar to trxB of *Mtb*. Both of these FHbs are functional as disulfide reductase in an oxygen-bound state, suggesting that their globin domain is acting as an oxygen sensor to regulate its functional activity. Generally, thioredoxin and thioredoxin reductase together with NADPH constitute a redox complex in which thioredoxin reductase catalyzes the electron transfer from NADPH to thioredoxin *via* FAD. The reduced thioredoxin with a dithiol/disulfide active site (CXXC) then reduces disulfide linkage and also serves as an electron donor in different metabolic processes. The globin domain in *Mtb*FHb appears to accept electrons from the reductase and may donate electrons to different cellular processes, very similar to the Trx/TrxR hybrid protein of *M. leprae* (Wang et al., 1998). Although the reductase domain of *Mtb*FHb carries three cysteine residues, it lacks a conventional CXXC active site. Mutation in Cys^188^ did not alter disulfide reductase activity of *Mtb*FHb, and this cysteine is not conserved in *Ms*FHbII as well as in other homologs of *Mtb*FHb in mycobacteria, which indicated that it may not be involved in disulfide reductase activities of these FHbs. Two other cysteine residues, Cys^289^ and Cys^360^, of *Mtb*FHb are fully conserved among type II FHbs, and mutation in any one of these residues appeared detrimental for the protein expression. It is quite likely that these two cysteines are crucial for the conformational stability and functional activity of these FHbs as a disulfide reductase by remaining close enough to maintain functional conformation and create an active site dithiol of the protein. This assumption was substantiated when Cys^289^ and Cys^360^ residues in the MtbFHbcys^188ala^ mutant were alkylated by IMA, which inhibited its NADPH-dependent DTNB reduction. These results demonstrated that Cys^289^ and Cys^360^ residues are important for the function of *Mtb*FHb as a disulfide reductase. It has been observed earlier that the mere presence of CXXC motif may not ensure a thioredoxin reductase-like property and distantly placed cysteines may also participate in forming an active site (Alam et al., 2007). Since *Mtb*FHb carries two distinct overlapping FAD-binding sites and FAD binds with the reductase domain of *Mtb*FHb in 1:1 stoichiometry (Gupta et al., 2012), it can be envisaged that these co-factor binding sites are utilized one at a time. It is quite intriguing how these sites and co-factors are selected for a specific reductase activity. *Mtb*FHb catalyzes the oxidation of D-lactate into pyruvate in a FAD-dependent manner having specific D-lactate oxidation activity as 28.3 µM/min/mg (Gupta et al., 2012). Our experimental data suggest that D-lactate creates a conformational change and attenuates NADPH-dependent disulfide reductase activity of *Mtb*FHb, changing its specificity towards reduction of di-mycothiol. Raman spectroscopic studies on *Mtb*FHb have shown that a structural change in the heme domain of the protein induces significant conformational changes within the reductase domain and inter-domain interactions are required for the stabilization of the sixth ligand within the heme active site (Gupta et al., 2012). Thus, it is likely that the D-lactate-mediated conformational change in *Mtb*FHb abrogates its interactions with NADPH and changes its substrate specificity from general dithiol to di-mycothiol. This is supported by the observation that the synthetic analog of mycothione (des-myoinositol) gets reduced by *Mtb*FHb only in the presence of D-lactate but not in the presence of NADPH. Mycobacteria carry millimolar concentration of mycothiol as the low-molecular-weight thiol, which is maintained in the cell in a reduced state by a mycothiol disulfide reductase (Mtr) that uses NADPH as an electron donor; however, its catalytic activity for mycothiol disulfide is one to two orders slower than other disulfide reductases (Patel and Blanchard, 1998), which may not be sufficient to maintain the level of reducing environment in the cell during rapid oxidation of mycothiol under severe oxidative stress that *Mtb* faces within the macrophagic environment (Shastri et al., 2018). Thus, the NADPH-dependent disulfide reductase activity of *Mtb*FHb can participate in conjunction with thioredoxin reductase and Mtr to create a robust antioxidant system that can play a vital role in protecting the viability and maintenance of redox balance by reduction of oxidized mycothiol and its recycling during intracellular infection when *Mtb* gets exposed to copious amounts of reactive oxygen and nitrogen species.

The dual function of *Mtb*FHb as NADPH-dependent disulfide reductase and D-lactate-dependent mycothione reductase appears unique and may be highly advantageous when *Mtb* gets exposed to a strong oxidative burst during macrophage infection. *Mtb*FHb carries high-affinity lipid binding sites and remains associated with membrane lipids (Gupta et al., 2012). Under high oxidative stress, membrane and cell wall lipids are among the most significant targets of oxidative damage. Since the lipid content of the cell envelope of *Mtb* is unusually high, decomposition of unsaturated fatty acids of *Mtb* cell wall into toxic methylglyoxal may generate D-lactate as a by-product of lipid peroxidation (Rachman et al., 2006), which may be highly toxic due to increased OH+ generation (Ali et al., 2000). It has been shown that the level of methylglyoxal increases significantly during mycobacterial infection of macrophages (Rachman et al., 2006). It may result in the accumulation of D-lactate in the cell as *Mtb* lacks a gene for the D-lactate dehydrogenase (Pinchuk et al., 2009). No functional D-lactate dehydrogenase activity has been demonstrated so far in *Mtb* that can metabolize accumulated D-lactate. Therefore, the proximity of *Mtb*FHb with the cell membrane and its D-lactate-dependent mycothiol disulfide reductase activity may be highly advantageous for the protection of cell wall integrity and maintenance of reducing environment under high oxidative stress. This is supported by the observation that *Mtb*FHb expressing cells of *M. smegmatis* are less prone to lipid peroxidation and more resistant to oxidative stress as compared to control cells. Additionally, expression of *Mtb*FHb in *Mtb*FHbII deleted mutant of *M. smegmatis* reduces lipid peroxidation and provides enhanced protection against different oxidants. During oxidation of D-lactate by *Mtb*FHb, the oxygen bound heme iron gets reduced and converted to Fe^2+^ state, suggesting that the heme domain is acting as an electron acceptor. Since *Mtb*FHb remains associated with the cell membrane, it is likely that it donates electrons to the menaquinone to feed the electron transport and use it as an electron sink for the removal of toxic D-lactate, generated during membrane lipid peroxidation. The H_2_O_2_ scavenging ability of pyruvate (Biagini et al., 2001; Guarino et al., 2019), produced during oxidation of D-lactate, may also contribute to the antioxidant activity of *Mtb*FHb.

It is interesting to note that besides *Mtb*, only two virulent mycobacteria, *M. avium* and *M. africanum*, have D-LDH-type FAD-binding motifs similar to *Mtb*FHb, whereas it is absent or mutated in other mycobacteria (**Figure S1**). It is likely that fast removal of D-lactate, accumulated due to membrane lipid peroxidation, is required for highly virulent mycobacteria to protect their membrane integrity and survival within the intracellular environment where large amounts of reactive oxygen and nitrogen species are produced. This is supported by the observation that the transcriptional activity of Rv0385 is induced several folds in *Mtb* during oxidative stress (Gupta et al., 2012) when accumulation of D-lactate may occur in the cell. Although *Ms*FHbII also carries overlapping D-LDH-type FAD-binding sites similar to *Mtb*FHb, the reduction of heme is extremely slow in the presence of D-lactate. Sequence composition of one of the intergenic regions of the LDH-type FAD-binding motif of *Ms*FHbII is different from *Mtb*FHb and carries non-polar residues that may affect solvent accessibility and the interactions of protein co-factor with the electron donor (Dym et al., 2000), thereby reducing its efficiency of electron transfer. It is likely that NADPH-dependent disulfide reductase and reduced efficiency of D-lactate oxidation by non-pathogenic mycobacteria like *M. smegmatis* may be sufficient to minimize their stress level.

Taken together, the present study provides the first report on dual function of *Mtb*FHb as a NADPH-dependent disulfide reductase and D-lactate-dependent mycothione reductase, which is modulated by two distinct overlapping FAD-binding sites. The D-lactate-dependent di-mycothiol reductase activity of *Mtb*FHb, observed during this study, appears unique and may be highly advantageous for *Mtb*. Under highly oxidative environment, when D-lactate accumulates due to excessive lipid peroxidation and rapid oxidation of mycothiol occurs, the substrate specificity of *Mtb*FHb gets diverted towards di-mycothiol, possibly due to conformational change in *Mtb*FHb by D-lactate that creates an active site that is highly specific for the reduction of di-mycothiol. It may provide a dual advantage to *Mtb* during exposure of highly oxidative environment by eliminating the toxicity of D-lactate accumulation in the cell, simultaneously maintaining the reducing environment of cell by facilitating reduction of mycothiol disulfide. Thus, *Mtb*FHb along with *Mtr* can constitute a robust system for the protection of *Mtb* within the oxidative environment, simultaneously balancing the redox environment of the cell during pathogenesis.

## Data Availability Statement

The original contributions presented in the study are included in the article/**Supplementary Material**. Further inquiries can be directed to the corresponding author.

## Author Contributions

KD conceived and designed the project. KD, RJ, and AK provided the resources and analyzed the data. NT, MH, and AC carried out experimental work. AS synthesized the des-*myo*-inositol disulfide mycothione and performed LC-MS analysis. MS was written by KD. All authors contributed to the article and approved the submitted version.

## Conflict of Interest

The authors declare that the research was conducted in the absence of any commercial or financial relationships that could be construed as a potential conflict of interest.

## Publisher’s Note

All claims expressed in this article are solely those of the authors and do not necessarily represent those of their affiliated organizations, or those of the publisher, the editors and the reviewers. Any product that may be evaluated in this article, or claim that may be made by its manufacturer, is not guaranteed or endorsed by the publisher.
