# Supplementary material for: New Insights Into the Function of Flavohemoglobin in Mycobacterium tuberculosis: Role as a NADPH-Dependent Disulfide Reductase and D-Lactate-Dependent Mycothione Reductase
Source: Front Cell Infect Microbiol. 2022 Feb 10;11:796727. doi: 10.3389/fcimb.2021.796727 (PMC8883573; doi:10.3389/fcimb.2021.796727)
Supplement: Supplementary file 1 [file Presentation_1.pdf]

New Insights Into the Function of Flavohemoglobin in *Mycobacterium tuberculosis*: Role as a NADPH-Dependent Disulphide Reductase and D-Lactate-Dependent Mycothione Reductase

Naveen Thakur[1], Amar Nath Sharma[1], Mangesh Dattu Hade[1], Ajay Chhaya[2], Ashwani Kumar[1] Ravinder Singh Jolly[1] and Kanak L. Dikshit[1,2]

[1]CSIR-Institute of Microbial Technology, Sector 39A, Chandigarh 160036, India
[2]Department of Biotechnology, BMS Block, Sector 25, Panjab University, Chandigarh 160014, India

Corresponding author: Kanak L Dikshit
E.mail: kanakdikshit@pu.ac.in

SUPPLEMENTARY MATERIAL

Experimental Procedures

List of primers used for the construction of the *fhb* gene knockout strains of mycobacteria

Primer sets used for the construction of *fhb* gene knock out strain of *Mtb*

Spe1upstream Forward -5’-GTACATGGCACTAGTTGCTTGCGGTATTCGTCC-3’

Swa1 upstream Reverse – 5’-GGAGCCAATTTAAAT GCAAACCAATGGGCATAG-3’

Pac I Downstream Forward- 5’-GTGCTCGTTAATTAAACCAAGGCCGCCCTGATCG-3’

Nsi I Downstream reverse – 5’-GTAGCTGCCATGCAT CGACAAGTTGCCAGCAACC-3’

Primer sets used for the construction of *fhb*II gene knock out strain of *M. smegmatis*

Spe1upstream Forward – 5’-GTACATGGCACTAGTGGCGTTACCCTCACATTC-3’

Swa1 upstream Reverse – 5’-GGAGCCAATTTAAATGGCGCTGCTCGGCCATCTCGG-3’

Pac I Downstream Forward- 5’-GTGCTCGTTAATTAAGGGACGCTCGCGGACGTCG-3’

Nsi I Downstream reverse – GTAGCTGCCATGCATGTCATGGTCGACGAATTG

Primer sets used for the construction of cysteine mutants of *Mtb*FHb

Primer set for *Mtb*FHb ^{cys188ala} mutant

5’- ATT CTG ATC GCC GGT GGA CCG GCC ATG GTC -3’

5’- CGG TCC ACC GGC GAT CAG AAT CTG CCG ATC -3’

Primer set for *Mtb*FHb ^{cys299ala} mutant

5’- CGC TAT GCC GCC GAA CTC TAC GAC CTG -3’

5’- GTC GTA CAG TTC GGC GGC ATA GCG TGC TCC-3’

Primer set for *Mtb*FHb ^{cys360ala} mutant

5’-GTT CCG CAA GCC GGG CGC CGG TGG CGA TAT CTC TCG-3’

5’-CCA CCG GCG CCC GGC TTG CGG AAC CAC GTT-3’

Primer set for qRT-PCR of Rv0385 (*Mtb*FHb)

5'-CTTCTATGCCCATTGGTTTGCC-3'

5'- GTTGCACAGCATGACCCAACC-3'

Primer set for qRT-PCR of MS_MEG0719 (*Ms*FHbII)

5'-CCGCGACTTCTACACCAACTG-3'

5'- GACAACGCAGTGTGGCATAAAC-3'

EXPERIMENTAL PROCEDURES

Chemical synthesis of *des-myo-inositol-mycothiol*

Materials:

All reagents, unless mentioned otherwise, were purchased from Sigma-Aldrich India Ltd. *N*-α-Fmoc-*S*-acetamidomethyl-L-cysteine pentaflurophenyl ester was purchased from Santa Cruz Biotechnology. Experiments were performed at 25 ^{o}C unless otherwise specified. ^{1}H NMR spectra were obtained at Jeol ECX 300 MHz spectrometer at 25 ^{o}C and referenced to TMS (0.0 ppm) or the residual solvent peak (H_2O, 4.66 ppm). LC-MS was done using Agilent Technologies G1316C and chromatography was performed on a reverse-phase column (Agilent C18, 2.1×50 mm; 1.8-Micron; ZORBAX Eclipse plus, USA). LC-MS analysis was conducted on Agilent G6550A MSQTOF. The whole output of column was introduced into the ESI-MS probe. The source voltage for electrospray was 3.5 kV and the fragmentation voltage was 150 V. The compound 5 was prepared following earlier published procedures (Patel and Blanchard, 1998) with slight modifications.

Chemical synthesis of des-myo-inositol-mycothiol (synthesised following the published procedure of Patel and Blanchard, 1998)

1. **Synthesis of *N*-α-Fmoc-S-acetamidomethyl-L-cysteinyl-2-amino-2-deoxy-α-D-glucopyranoside (formula 3, Fig S4A)**

17.25 ml of a 1M NaOH (0.69 g, 17.29 mmol) solution was added slowly in a dropwise manner by syringe to D-glucosamine hydrochloride (formula **1** Fig S4A, 3.71g, 17.29 mmol). The mixture was stirred for 10 minutes. Water was removed in *vacuo* and resulting D-glucosamine and NaCl slurry was used for further reaction. Hydroxybenzotriazole, HOBt (930 mg, 6.91

mmol) was added to solution of *N*-α-Fmoc-*S*-acetamidomethyl-L-cysteine pentaflurophenyl ester (formula **2** Fig S2A, 2 g, 3.45 mmol) in dimethylforamide (DMF). This solution was transferred to slurry of D-glucosamine and NaCl, and contents stirred under a dry nitrogen environment for 2 hours. The solvent DMF was removed in *vacuo* and coupling product **3** was precipitated by addition of water (100 mL). The precipitate was collected by filtration and washed with water to remove excess of D-glucosamine, NaCl, HOBt, pentafluorophenol, and residual DMF. The resultant product was dried under *vacuo* to yield compound of formula **3** **(Fig S4A)** as a white solid (1.5 g, 75%). TLC of the product indicated the presence of a single compound (1:1 hexane/EtOAc, R_f 0.1).

2. Synthesis of *N*-acetyl-S-acetamidomethyl-L-cysteinyl-2-amino-2-deoxy-α-D-glucopyranoside (product 4, Fig S4A)

A solution of Fmoc coupled product (formula 3 Fig S4A,1.4 g, 2.43 mmol) and piperidine (0.5ml) in DMF (5 ml) was stirred for 2 hours. The solvent was evaporated in *vacuo* and cleaved dibenzofulvene was precipitated with water (100 ml). The precipitate was separated by using a 0.22 µm cellulose filter (Millipore). The aqueous part containing amine and residual piperidine and DMF was evaporated in *vacuo*. The amine was sticky solid and used for *N*-acetylation without purification. The amine was dissolved in aqueous K_2CO_3 (265 mg, 1.61 mmol, 3 ml) andthen acetic anhydride (90 mg, 0.89 mmol) was added to this solution. The solution was stirred for 2 hours and solvents evaporated in *vacuo*. The solid residue was treated with EtOH to precipitate K_2CO_3. After precipitation, ethanol was evaporated in *vacuo* and solid residue was purified by preparative HPLC [Merck C18, 250×10 mm, detection UV at 210 nm, 0 to 50 min (100% H_2O, 3ml /min), 50-75 min (100% MeOH, 3ml/min), t_R: 21.83 min] to yield compound of formula **3,** Fig S4A as white powder, which was characterised by NMR. ^{1}H NMR (**Fig S4B**, 300 MHz, D_2O): δ 5.07-5.06 (d, 1H), 4.48-4.43 (m, 1H), 4.27-4.14 (m, 2H), 3.89-3.30 (m, 6H), 3.01-2.92 (dd, 2H), 2.09 (s, 3H), 2.05 (s, 3H). This spectrum contains two isomers due to mutarotation of the glucosamine pyranose ring in D_2O.

3. Synthesis of *N*-acetyl-L-cysteinyl-2-amino-2-hydroxy-α,β-D-glucopyranoside disulphide (product 5, Fig S4A)

Compound of formula **4, Fig S4A** (110 mg, 0.27 mmol) was added to solution of anisole in trifluoroacetic acid (TFA) at 4 ^{o}C and the contents magnetically stirred in an oven-dried Schlenk flask under an argon atmosphere. Tallium (III) trifluoroacetate (160 mg, 0.28 mmol) was added and stirring continued for further 2 hours. Solvent was evaporated in *vacuo* and

residue triturated with ether (50 ml). The ether layer was decanted and this process was repeated 2 more times with ether. The ether layer was evaporated in *vacuo* and residue was purified by preparative HPLC using same procedure which was used **in step 2** to yield 29 mg of white solid (yield 25%). The product (formula **5, Fig S4A)** was characterised by NMR and mass spectroscopy. ^{1}H NMR (**Fig S4C**, 300 MHz, D_2O): δ 5.09-5.07 (d, 1H), 4.771H) (overlap with D_2O), 3.87-3.31 (m, 6H), 3.16-2.79 (dd, 2H), 1.94 (s, 3H).Due to presence of diastereomers, the anomeric proton did not integrate to one; see explanation above for **4**. LC-MS (Fig [Agilent C18, 2.1 ×50 mm, 0 min (100% H_2O), 8.0 min (98% H_2O, 2% CH_3CN) 10 min (80% H_2O, 20% CH_3CN), 12 min (60 % H_2O, 40% CH_3CN), 14.0 min (40 % H_2O, 60% CH_3CN), 16.0 min (20% H_2O, 80% CH_3CN,) 18.0 min (100% CH_3CN) , 0.4 ml/min, t_R: 3.81-4.20 min] ESI-MS (Observed): m/z 628.17 $(M-H_2O)^+$, (Calculated) 628.17 $(M-H_2O)^+$

Reference

1. Patel, M. P., & Blanchard, J. S., 1998. Synthesis of Des-myo-inositol mycothiol and demonstration of a mycobacterial specific reductase activity. *Journal of the American Chemical Society*, ***120***, 11538-11539).

Suplementary Figures

```
                                                        B9B10          CD1CD2    E7
M.tuberculosis    MGLEDRDALRVLQNAFKLD--------DPELVRRFYAHWFALDASVRDLFPPDMGAQRAA
M.smegmatis       ----------MLRAAVDPAND------SDPLIRDFYTNWFAADLSVRDLFPPEMAEQRRV
M.avium           MSLEDADALRVLRDAFAPCD-AGRPGDSGDLVHRFYTHWFALDPSVRDLFPPEMGAQRVA
M.africanum       MGLEDRDALRVLQNAFKLD--------DPELVRRFYAHWFALDASVRDLFPPDMGAQRAA
M.marinum         MGLEDRDALRVLRDAFAPQPDLDHKTQSSELVRSFYTNWFSLDSSVRDLFPPEMSGQRAA
M.ulcerns         MGLEDRDALRVLRDAFAPQPDLDHKTQSSELVRSFYTNWFSLDSSVRDLFPPEMSGQRAA
M.gordonae        MGLEDRDALQVLQDAFAPG--------STELVHRFYDHWFALDTSVRDLFPPEMSGQRAA
M.gilvum          --------MRILREAFEPGPG------SERLVGEFYTRWFAADLTARDLFPPDMAGQRQV

                                                   F7F8       G5
M.tuberculosis    FGQALHWVYGELVAQRAEEPVAFLAQLGRDHRKYGVLPTQYDTLRRALYTTLRDYLGHPS
M.smegmatis       FAHALTWLFGELIAQRAEDPITFLAQLGRDHRKYGVTQQHYDSMQSALYGALKARLA---
M.avium           FGHALHWVYGELVARRAQEPVAFLAQLGRDHRKYGVLPRHYQTLGRALLATLRSHLD---
M.africanum       FGQALHWVYGELVAQRAEEPVAFLAQLGRDHRKYSVLPTQYDTLRRALYTTLRDYLGHPS
M.marinum         FTRALHWVYSELVAQRAEEPIAFLAQLGRDHRKYGVQPTQYETLRRALQTTLRSHLG---
M.ulcerns         FTRALHWVYSELVAQCAEEPIAFLAQLGRDHRKYGVQPTQYETLRRALQTTLRSHLG---
M.gordonae        FAQAMHWLYGELVAQRADEPVAFLAQLGRDHRKYGVLPAHYQTLRRALYATLRSHLG---
M.gilvum          FAHAMRWLCDELIAQRAEEPVAFLAQLGRDHRKYGVTPEHYAGLQDALLAAVRTTLT---

M.tuberculosis    RGAWTDAVDEAAGQSLNLIIGVMSGAADADDAPAWWDGTVVEHIRVSRDLAVARLQLDRP
M.smegmatis       -DRWTDRLAAATRDAVALFIGVMRGAADAEESPAYCDGTVVETHRLTRDVSVIRLQLDEP
M.avium           -AAWTDAVEDAARTSLNLITGVMSGAADADEGPAWWDGTVIGHLRVSRDLAVVRLRLDQP
M.africanum       RGAWTDAVDEAAGQSLNLIIGVMSGAADADDAPAWWDGTVVEHIRVSRDLAVARLQLDRP
M.marinum         -SSWTDSVARAAEQSLNLIVGVMSGAADSEDGPAWWDATVIEHIPASRDLAVIRLQLDHP
M.ulcerns         -SSWTDSVARAAEQSLNLIVGVMSGAADSEDGPAWSDATVIEHIPASRDLAVIRLQLDHP
M.gordonae        -SFWTDAVQEAADQSLNLFAGVMSGAADSDDAPAWVDGTVIEHNRVSRDLAVVRLHLDRP
M.gilvum          -DRWDARLEEAATDVVTLAVGVMSGAGEAETAPPFCDGTVLEHLRPTRDVSVIRLKMDHH

M.smegmatis       LFYHSGQYVTVQVPQWPRRWRYLSPAIPSDRSGAIEFHVRSVTGGMVSTAIVNETRHGDR
M.avium           MPYHPGQYVNVQVPQCPRRWRYLSPAIPADPGCGIEFHVRLVPGGLVSTAIVNETRPGDR
M.africanum       LHYYPGQYVNVHVPQCPRRWRYLSPAIPADPNGRIEFHVRVVPGGLVSNAIVGETRPGDR
M.marinum         MPYHPGQYVNVQVPQCPRRWRYLSPAIPADLEGRIEFHVRVVPGGLVSTAMVNETRTGDR
M.ulcerns         MPYHPGQYVNVQVPQCPRRWRYLSPAIPADLEGRIEFHVRVVPGGLVSTAMVNETRTGDR
M.gordonae        LHYHPGQYVNVQIPQCPRRWRYLSPAIPADPGGGIEFHVRVVPGGLVSNAIVGETRPGDR
M.gilvum          IDYHAGQYVSVQVPQWPRRWRFLSPAIPADPQGYVEFHVRSVAGGMVSTTILGETRPGDR

                              LDH FAD motif1     LDH FAD motif 2
M.tuberculosis    WRLSGPHGAFRVDRDGGDVLMVAGSTGLAPLRALIIDLSRFAVNPRVHLFFGARYACELY
M.smegmatis       WRVSSPHGALEVNRSGEDVLMVAGSTGLAPLRALIMDMTLHAENPRVHLFFGGRFPCDLY
M.avium           WRLSSPHGGLRVDRDGGDVLMVAGSTGLAPLRALIMDLSRFAVNPRVHLFFGARYRCELY
M.africanum       WRLSGPHGAFRVDRDGGDVLMVAGSTGLAPLRALIIDLSRFAVNPRVHLFFGARYACELY
M.marinum         WRLASPHGGLHVDRTGGDVLMVAGSTGLAPLRALIILDMGRFVENPRVHLFFGARYFCELY
M.ulcerns         WRLASPHGGLHVDRTGGDVLMVAGSTGLAPLRALIILDMGRFVENPRVHLFFGARYFCELY
M.gordonae        WRLSGPHGGLRVDRDGGDVLMVAGSTGLAPLRTLIMDLSRFGVNPRVHLFFGARYRCELY
M.gilvum          WRLAAPHGAMHVDREAGDVLMVAGSTGLAPLRALIMDMCRFAANPRVHLFFGAKYPCELY
```

*trx*R like FAD-motif

```
M.tuberculosis    DLPTLWQIAAHNPWLSVSPVSEYNGDPAWAADYPDVSAPRGLHVRQTGRLPDVVSRYGGW
M.smegmatis       DLKTLWTIASTNPWLSVTPVSEYSTDPPWARDYPDPTPPRGLHVRQTGTLADVVTRYGNW
M.avium           DLPTLWQIASHNPWLSVSPVSEYRADPPWAADYPDVTPPRGLHVHQTGRLPEVVTKYGGW
M.africanum       DLPTLWQIAAHNPWLSVSPVSEYNGDPAWAADYPDVSAPRGLHVRQTGRLPDVVSRYGGW
M.marinum         DLRTLWQVAAHNPWLSVSPVAEYKLDPSWATDYPDVSPPRGLHVRQTGRLAEVVTNYGNW
M.ulcerns         DLRTLWHVAAHNPWLSVSPVAEYKLDPSWATDYPDVSPPRGLHVRQTGRLAEVVTNYGNW
M.gordonae        DLRTLWQVAAHNPWLSVSPVSEYNNDPAWAADYPDVSPPRGLHVRQTGRLPDVVTRYGAW
M.gilvum          DLPTLWEVASMNPWLSVTPVSEFDFDPPWAAEYPDNRPPRGLHVRQTGRLDEVVTRYGSW
```

```
                         NADPH :Adenine              Glu394
M.tuberculosis      GDRQILICGGPAMVRATKAALIAKGAPPERIQHDPLSR-
M.smegmatis         GDRQILICGGPQMVEATKAALIAKGAPPERIQHDPLTAR
M.avium             GDRQILICGGPRMVAATKAALIAKGAAAQRIQHDPLSR-
M.africanum         GDRQILICGGPAMVRATKAALIAKGAPPERIQHDPLSR-
M.marinum           GDRQILICGGPAMVRTTKAALIAKGAPRERIQHDPLP--
M.ulcerns           GDRQILICGGPAMVRTTKAALIAKGAPRERIQHDPLP--
M.gordonae          GDRQILICGGPQMVRATKAALIAKGAPPELIQHDPLSR-
M.gilvum            GDRQILVCGRPEMVAATRSALIAKGAPAERIQHDPLGN-
```

Fig. S1. Sequence alignment of *Mtb*FHb with type II FHbs of mycobacteria. Conserved residues in the heme and reductase domains are marked and highlighted in yellow color. Thioredoxin reductase type FAD binding site is highlighted in grey and conserved sequences [**D(x)8GxxP**) are shown in red. D-LDH-type FAD finding sites 1 and 2 **[A(x)7AxN**, **G(x)7 GS]** are shown in green outlined boxes. Conserved residues are shown in orange and mutated residues within the FAD binding motif are underlined.

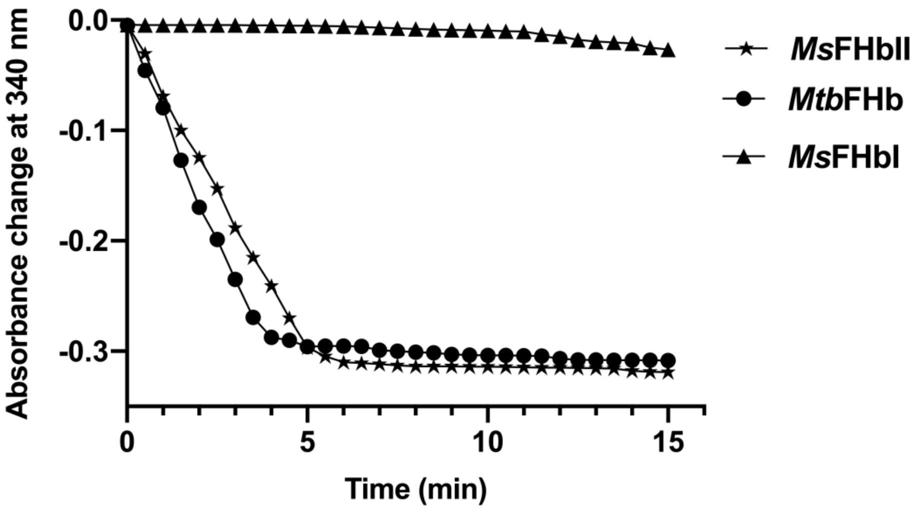

FIGURE S2 . NADPH oxidase activity of type I (*Ms*FHbI) and type II FHbs of *M. smegmatis* (*Ms*FHbII) and *Mtb* (*Mtb*FHb). The reaction was set up in 0.5 ml containing NADPH (250 uM), 0.5 M potassium phosphate buffer (pH 7.2) and 1 mM EDTA. Baseline level of NADPH oxidation was set before addition of the protein in the reaction mixture. Oxidation of NADPH was monitored spectrophotometrically at 340 nm after adding 8 µM of protein (*Ms*FHbI, *Ms*FHbII or *Mtb*FHb) in the reaction mixture.

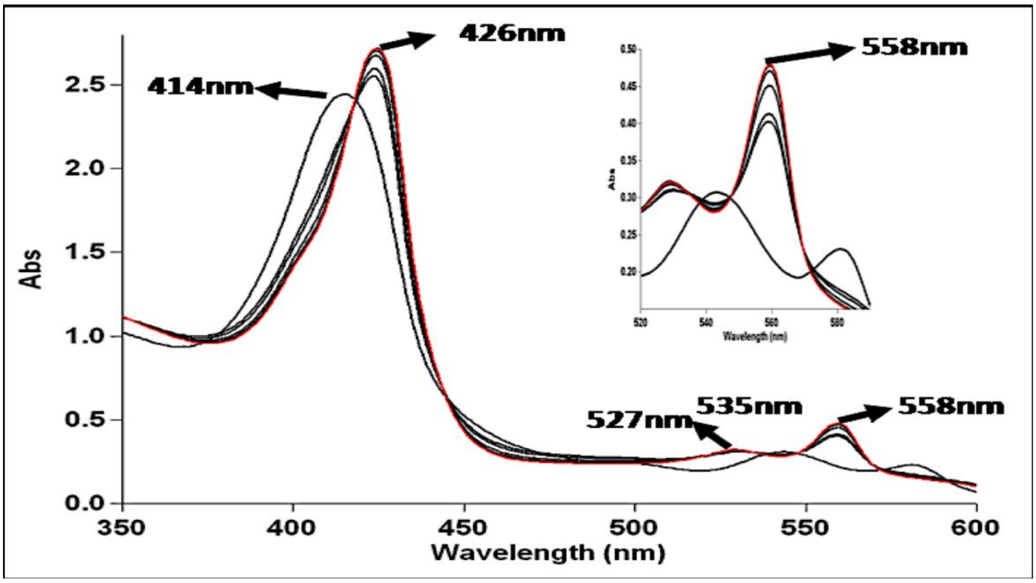

FIGURE S3. D-lactate mediated reduction of oxy-*Mtb*FHb. Absorption spectra of *Mtb*FHb in the native (oxy-*Mtb*FHb, showing 414 Soret peak) and after reduction with D-lactate (red line). Scans were taken every after five seconds. Inset shows the enlarged view of the spectra from 520 nm to 600 nm

4A

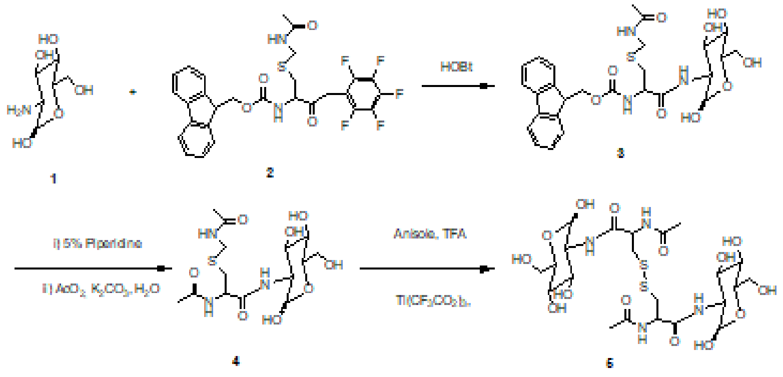
HOBt
i) 5% Piperidine
ii) AcO2, K2CO3, H2O
Anisole, TFA
Tl(CF3CO2)3,
1
2
3
4
5

4B

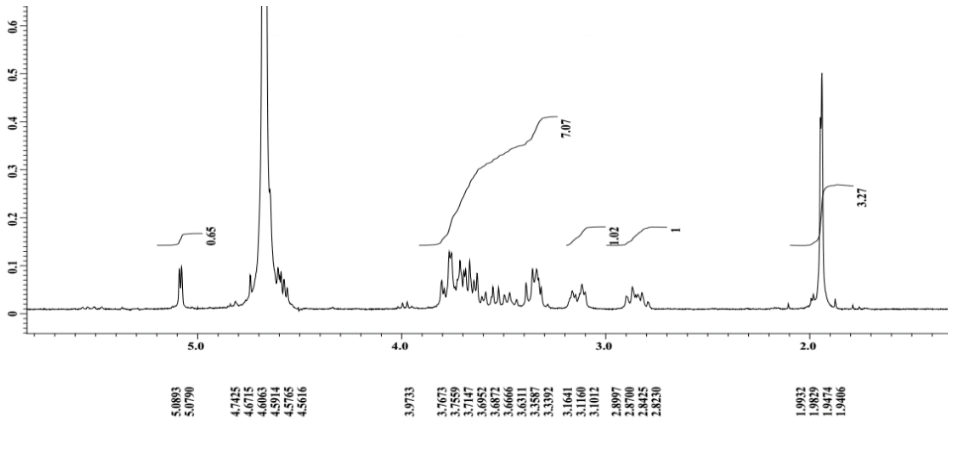
0.65
7.07
1.02
1
3.27
5.0
4.0
3.0
2.0
5.0893
5.0790
4.7425
4.6715
4.6063
4.5914
4.5765
4.5616
3.9733
3.7673
3.7559
3.7147
3.6952
3.6872
3.6666
3.6311
3.3587
3.3392
3.1641
3.1160
3.1012
2.8997
2.8700
2.8425
2.8230
1.9932
1.9829
1.9474
1.9406

4C

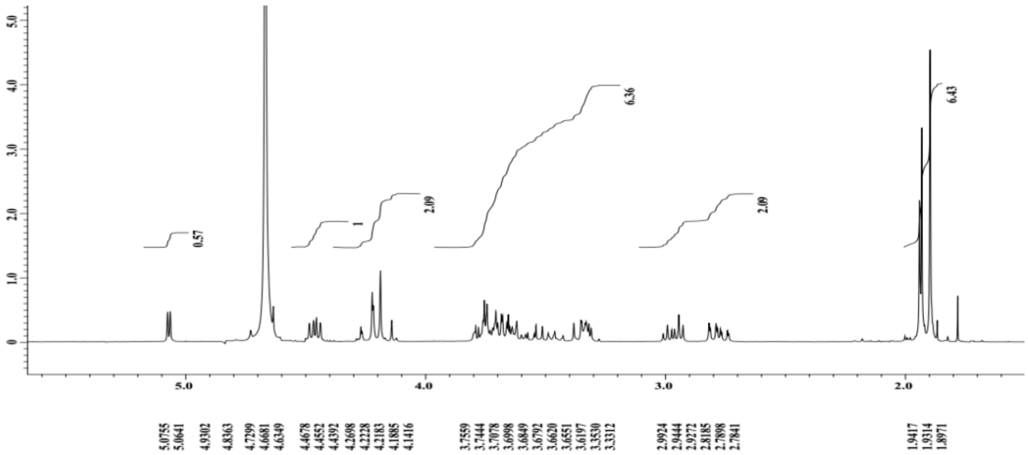

FIGURE S4. Chemical synthesis of des-myo-inositol mycothiol. (A) Steps showing preparation reactions for the synthesis of des-myo-inositol- mycothiol . **B**. ^{1}HNMR spectra of N-α-Fmoc-S-acetamidomethyl-L-cysteinyl-2-amino-2-deoxy-α-D-glucopyranoside and **C**. ^{1}HNMR spectra of N-acetyl-L-cysteinyl-2-amino-2-deoxy-α-D-glucopyranoside

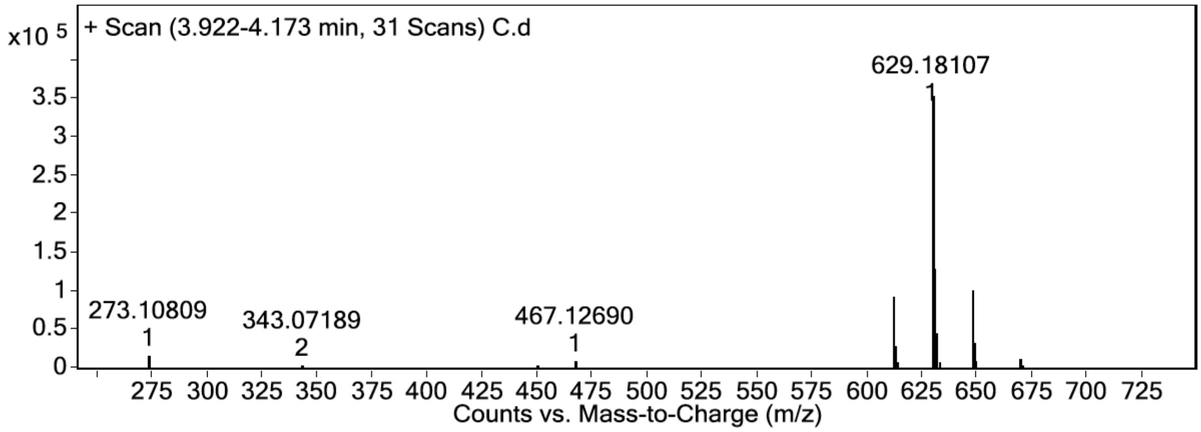

FIGURE S5. Mass spectrophotometric analysis of des-myoinositol (synthetic analog of mycothione) in the presence of NADPH. The assay was performed in 100 µl reaction mixture containing 50 mM HEPES (pH 7.6) and 0.1 mM EDTA. *Mtb*FHb (5µg each) was incubated individually with 100 µM des myo-inositol-mycothione along with 250 µM NADPH at 25°C for 5 min and the product was analysed via LC-MS

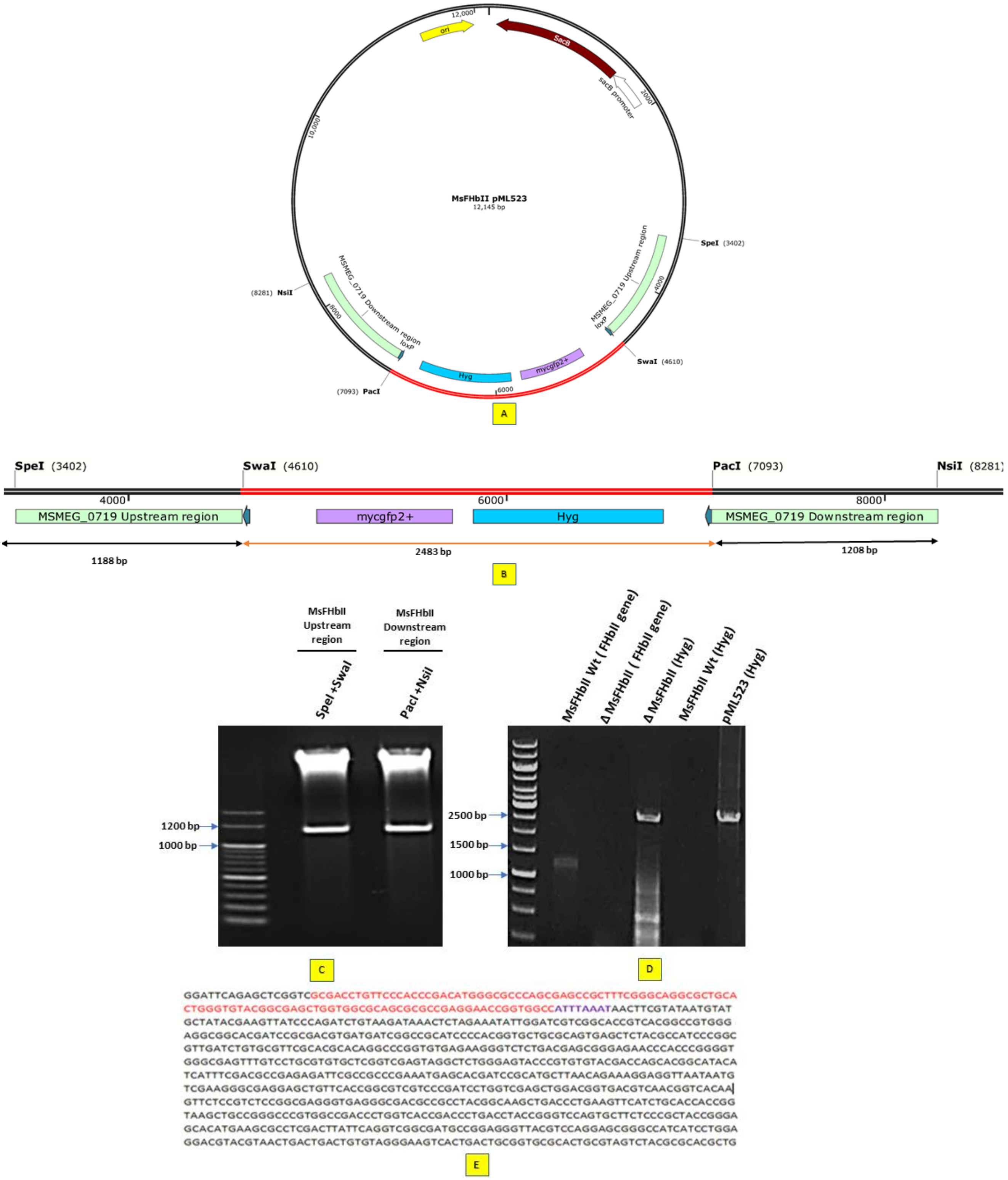

Fig S6. PCR analysis and confirmation of *fhb*II gene deletion in *M. smegmatis*. A: Full circular map of the *fhb*II gene knock out plasmid, Ms FHbII pml253 (pML523 ^{MsFHbII}). **B.** Schematic presentation of the region showing integration of upstream and downstream region

of the *fhb*II gene of *M. smegmatis* with mycgfp2+ and hygromycin cassette in pML523 ^{MsFHbII} **C**: Confirmation of upstream and downstream region of the *fhb*II gene of *M. smegmatis* in pML523 ^{MsFHbII} with gene specific restriction enzymes digestion. **D**: Confirmation of *fhb*II gene knockout in *M. smegmatis* by amplifying the *fhb*II gene specific primers and the mycgfp2+ - Hygromycin cassette specific primers using the genomic DNA of wild type and the *fhb*II gene knock out strains of *M. smegmatis*. **D**: Confirmation of *fhb*II gene deletion after nucleotide sequencing of PCR product generated from the genomic DNA of MsΔ*fhb*II (*fhb*II gene knock out *M. smegmatis*) using upstream and downstream primers of *fhb*II gene of *M.* s*megmatis.* Sequences in red are showing the upstream region of the *fhb*II gene lacking the coding sequences and the sequences of hygromycin gene.

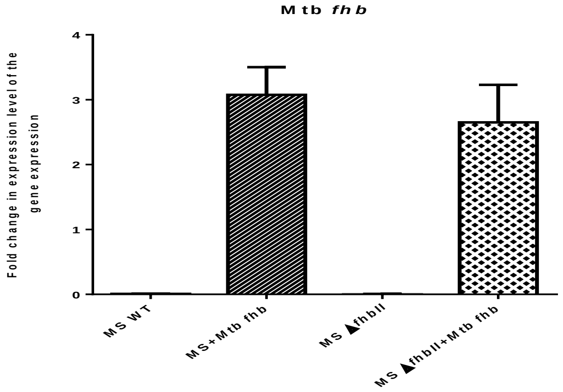

Fig. S7. Transcript analysis during expression of *Mtb fhb* gene in *M. smegmatis*. qRT-PCR analysis of the *Mtb fhb* gene transcript during overexpression of *Mtb fhb* gene expressing wild type and MSΔ*fhb*II strains of *M. smegmatis. M. smegmatis* cultures were grown aerobically till the $O.D_{600}$ reaches to 1.0 under the condition mentioned in experimental procedures. Thereafter, samples were withdrawn and analysed through qRT-PCR in triplicate. Relative fold changes in *Mtb fhb* gene expression were analysed for each biological replicate using Livak ($2^{-\Delta\Delta Ct}$) method and normalized with respect to housekeeping gene *sig*A. Statistical analysis was performed by one-tailed t-test using GraphPad Prism version 8.4.3 for Windows ($p < 0.01$).
